# Bioenergetic Heterogeneity in Mycobacterium tuberculosis Residing in Different Subcellular Niches

**DOI:** 10.1128/mBio.01088-21

**Published:** 2021-06-01

**Authors:** Ajit Kumar Akela, Ashwani Kumar

**Affiliations:** a Council of Scientific and Industrial Research, Institute of Microbial Technology, Chandigarh, India; b Academy of Scientific and Innovative Research (AcSIR), Ghaziabad, India; Yale School of Medicine

**Keywords:** Bioenergetics, ATP/ADP, metabolic state, Perceval HR, *Mycobacterium tuberculosis*, subcellular compartments, antimycobacterial drugs, intracellular bacteria, metabolic heterogeneity, metabolism, PHR-mCherry

## Abstract

ATP/ADP depicts the bioenergetic state of Mycobacterium tuberculosis (Mtb). However, the metabolic state of Mtb during infection remains poorly defined due to the absence of appropriate tools. Perceval HR (PHR) was recently developed to measure intracellular ATP/ADP levels, but it cannot be employed in mycobacterial cells due to mycobacterial autofluorescence. Here, we reengineered the ATP/ADP sensor Perceval HR into PHR-mCherry to analyze ATP/ADP in fast- and slow-growing mycobacteria. ATP/ADP reporter strains were generated through the expression of PHR-mCherry. Using the Mtb reporter strain, we analyzed the changes in ATP/ADP levels in response to antimycobacterial agents. As expected, bedaquiline induced a decrease in ATP/ADP. Interestingly, the transcriptional inhibitor rifampicin led to the depletion of ATP/ADP levels, while the cell wall synthesis inhibitor isoniazid did not affect the ATP/ADP levels in Mtb. The usage of this probe revealed that Mtb faces depletion of ATP/ADP levels upon phagocytosis. Furthermore, we observed that the activation of macrophages with interferon gamma and lipopolysaccharides leads to metabolic stress in intracellular Mtb. Examination of the bioenergetics of mycobacteria residing in subvacuolar compartments of macrophages revealed that the bacilli residing in phagolysosomes and autophagosomes have significantly less ATP/ADP than the bacilli residing in phagosomes. These observations indicate that phagosomes represent a niche for metabolically active Mtb, while autophagosomes and phagolysosomes harbor metabolically quiescent bacilli. Interestingly, even in activated macrophages, Mtb residing in phagosomes remains metabolically active. We further observed that macrophage activation affects the metabolic state of intracellular Mtb through the trafficking of Mtb from phagosomes to autophagosomes and phagolysosomes.

## INTRODUCTION

ATP/ADP is central to the bioenergetic state of cells (1, 2). ATP/ADP directly regulates several metabolic pathways such as glycolysis (3), the tricarboxylic acid (TCA) cycle, and respiratory flux of electrons (4). Low ATP/ADP levels stimulate the oxidation of the TCA cycle’s substrates through isocitrate dehydrogenase (5). This ratio is also a selective indicator of energy consumption and changes in energy metabolism in a cell (6). ATP/ADP homeostasis also plays a crucial role in determining the physiological state of Mycobacterium tuberculosis (Mtb) (7). Importantly, Mtb could shuttle between an actively replicating drug-susceptible state and a nonreplicating drug-tolerant state in response to environmental cues such as hypoxia, nitric oxide (NO), carbon monoxide, and nutrient starvation (8–11). Interestingly, these cues are associated with a downshift in the metabolism of Mtb, characterized by inhibition of respiration, accumulation of NADH, and depletion of ATP levels. Although the first-line antituberculosis (anti-TB) drugs isoniazid (INH) and rifampicin (RIF) are unable to kill Mtb in the nonreplicating state, a recently identified inhibitor of ATP synthase, bedaquiline (BDQ) (12), is capable of killing these metabolically quiescent cells, suggesting the importance of ATP synthesis in persister cells (13).

Given the significant role of ATP/ADP in regulating metabolism and bacterial physiology, several methods are employed to estimate ATP/ADP. Traditional methods rely upon the disruption of cells and measurement of the ensemble average of a population and ignore intrinsic cellular heterogeneity (14). Emerging literature suggests that even under growth-promoting conditions, some bacterial cells stochastically behave similarly to cells from the stationary phase, have depleted levels of ATP, and could tolerate bactericidal concentrations of antibiotics (15, 16). These studies delineate the importance of cellular heterogeneity in a population. Therefore, a reporter with spatial resolution is required to measure cellular ATP levels. For this purpose, firefly luciferase is widely used, and it utilizes ATP for the production of quantifiable luminescence. This enzyme is genetically encoded (17, 18). However, its use is limited due to weak signals. Furthermore, luciferase consumes cellular ATP, which could affect cellular physiology, and thus, it is not suitable for temporal measurements. Notably, the metabolic state and intracellular oxygen levels are known to interfere with luciferase bioluminescence (19). Furthermore, the bioluminescence of luciferase is influenced by the luciferase structure and assay conditions (20). To mitigate these problems, an array of genetically encoded biosensors were developed.

ATeam is a fluorescence resonance energy transfer (FRET)-based biosensor of ATP wherein the ATP-responsive epsilon subunit of the bacterial F_o_F_1_-ATP synthase is sandwiched between cyan fluorescent protein and yellow fluorescent protein (YFP) (21). The epsilon subunit of the bacterial F_o_F_1_-ATP synthase was inserted in enhanced green fluorescent protein (eGFP) for the engineering of QUEEN (22) or in superfolder green fluorescent protein (GFP) for the engineering of iATPSnFRs (23). In another approach, GlnK1, an ATP binding trimeric protein of the PII family, was fused with cpmVenus to create the sensor dubbed Perceval (24). However, Perceval suffered from saturation at low ATP/ADP (<5) and was thus reengineered to Perceval HR (PHR) through extensive mutagenesis in the ATP binding pocket of Perceval (25). PHR is capable of measuring a higher dynamic range of ATP/ADP levels (25). One of these genetically encoded FRET-based ATP sensors, ATeam, was also employed for tracking the metabolic state of Mycobacterium smegmatis (Msmeg) (26). Since mycobacteria possess autofluorescence (27), the optimal usage of this sensor in Msmeg required dilation of the fluorescence through a deletion mutation in coenzyme F_420_ encoded by the *fbiC* gene (26). However, the measurement of ATP/ADP levels in Mtb remains a daunting challenge.

Mtb is an intracellular pathogen. Mtb primarily infects macrophages and could reside in several intracellular compartments such as phagosomes, autophagosomes, phagolysosomes, and autophagolysosomes (28). However, the metabolic state of Mtb residing inside macrophages has remained poorly understood in the absence of suitable tools with spatial resolution. In this study, we have modified PHR to a new ATP/ADP ratiometric biosensor termed “PHR-mCherry.” This novel probe is capable of monitoring ATP/ADP of Mtb and Msmeg. We validated the PHR-mCherry sensor (ATP/ADP sensor) *in vitro* and *in vivo*. PHR-mCherry was then used to measure ATP/ADP in mycobacteria upon exposure to antibiotics or other physiologically relevant stresses. Importantly, we have studied the metabolic state of mycobacteria residing in different subvacuolar niches inside macrophages. We observed that Mtb cells residing inside phagosomes maintain high ATP/ADP levels. In contrast, Mtb cells residing inside phagolysosomes or autophagosomes face metabolic stress as depicted by reduced ATP/ADP levels.

## RESULTS

### Intrinsic mycobacterial autofluorescence prevents the use of a known ATP/ADP reporter, PHR.

Previously, we utilized the Peredox sensor (29) to engineer reporter strains of fast- and slow-growing mycobacterial species for spatiotemporal measurement of NADH/NAD^+^ (30, 31). We intended to create reporter strains of fast- and slow-growing mycobacteria using the PHR sensor on the same lines. The excitation spectrum of PHR with an emission maximum (Emi_max_) of 525 nm has two distinct peaks at 420 nm and 500 nm that increase in response to ADP and ATP, respectively (25). Thus, the 500/420 ratio depicts the ATP/ADP. We cloned the genetic region encoding the sensor PHR in pMV762 and transformed it into Mtb. An excitation spectrum was recorded using a fluorimeter with an Emi_max_ of ∼525 nm. Incidentally, we observed a weak peak at 420 nm and a sharp peak at 500 nm (see Fig. S1A and B in the supplemental material). To ensure that these peaks are due to ATP/ADP, we utilized the ATP synthase inhibitors BDQ and *N*,*N*-dicyclohexylcarbodiimide (DCCD). BDQ inhibits ATP synthesis by inhibiting ATP synthase, and DCCD also inhibits ATP synthesis by covalent binding with protonated carboxylates of the C-ring ion binding site of ATP synthase (32). Unfortunately, we did not observe a significant and consistent decrease in the 500/420 ratio in response to BDQ or DCCD, suggesting that the sensor PHR is not functional in Mtb (Fig. S1C). However, closer scrutiny of the data indicated that the 500-nm peak in the excitation spectrum decreased upon treatment of mycobacterial cells with both BDQ and DCCD (Fig. S1D). We observed similar spectral properties of PHR overexpressed in Msmeg using pMV762. Since Mycobacterium has strong intrinsic fluorescence with an excitation maximum (Ex_max_) at ∼420 nm and an emission maximum at ∼480 nm (27), we analyzed the autofluorescence of Msmeg and Mtb at an Emi_max_ of 525 nm (Fig. S1E and F). These observations suggest that the 420-nm peak observed in the Mtb cells’ excitation spectrum with PHR is due to autofluorescence. Given the requirement of a ratiometric probe for the normalization of expression and because only one of the two peaks responded to cellular ATP/ADP levels, we concluded that PHR is not useful for measuring ATP/ADP levels in mycobacterial cells.

10.1128/mBio.01088-21.1 FIG S1 Analysis of the capability of PHR for monitoring ATP/ADP in Mtb. (A) Excitation spectra of Mtb overexpressing PHR. (B) Emission spectra of Mtb overexpressing PHR. (C) Bar graph representing the 500/420 (ATP/ADP) ratio of Mtb cultures independently treated with BDQ and DCCD for 6 h. Data shown are expressed as means ± SEM and are illustrative of results from three independent experiments performed in triplicates. Data were plotted using GraphPad Prism software. (D) Excitation spectra of the PHR reporter strain of Mtb upon treatment with BDQ and DCCD. (E) Excitation spectra of M. smegmatis mc^2^155 at emission maxima of 530 nm. (F) Excitation spectra of Mtb H37Rv at emission maxima of 530 nm. Download FIG S1, TIF file, 0.9 MB. Copyright © 2021 Akela and Kumar. 2021 Akela and Kumar. https://creativecommons.org/licenses/by/4.0/ This content is distributed under the terms of the Creative Commons Attribution 4.0 International license.

### Reengineering of PHR for measurement of ATP/ADP in Mycobacterium.

**FIG 1:**
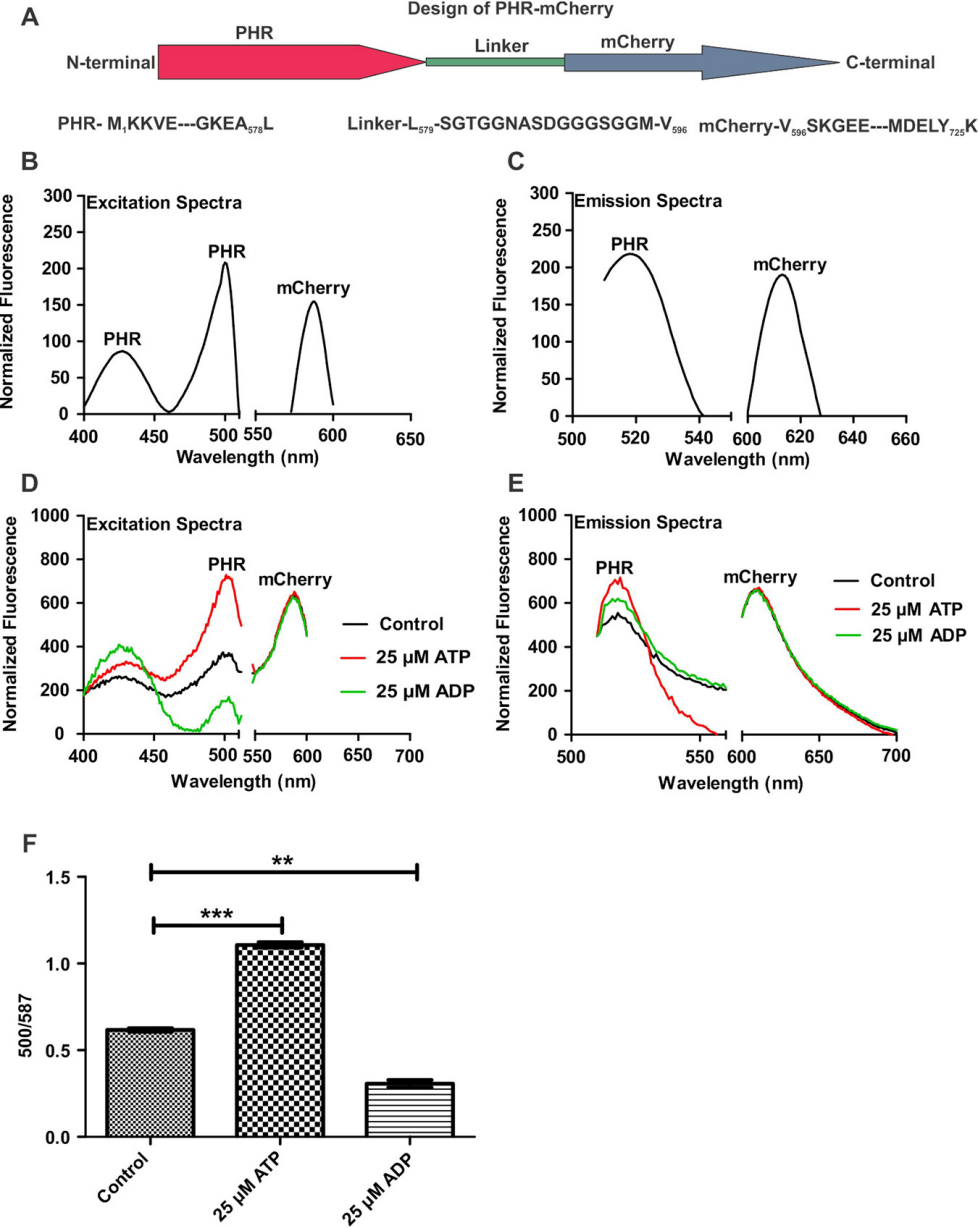
PHR-mCherry sensor responds to ATP/ADP levels. (A) Cartoon depicting the design of the new sensor protein PHR-mCherry. (B) PHR-mCherry was overexpressed in E. coli and purified using a Ni-NTA column. The excitation spectra were recorded with 10 μM PHR-mCherry. (C) Fluorescence emission spectra of 10 μM PHR-mCherry. (D) PHR-mCherry at 10 μM was treated with 25 μM ATP or ADP, and the excitation spectra were recorded. (E) Emission spectra of PHR-mCherry treated with ATP, ADP, and the no-treatment control. In panels D and E, red, green, and black spectra represent PHR-mCherry treated with ATP, PHR-mCherry treated with ADP, and PHR-mCherry alone, respectively. (F) Bar graph representing the ratiometric responses of sensor proteins to 25 μM ATP and 25 μM ADP. Data presented here are representative of results from three independent experiments performed in technical triplicates. Data were plotted using GraphPad Prism software and are presented as means (±SEM). The statistical significance of the data was determined by using a two-tailed unpaired *t* test. **, *P* < 0.01; ***, *P *< 0.001.

As described above, the major ATP/ADP-responsive excitation peak of PHR at 500 nm was amenable to cellular ATP/ADP. To utilize this property of PHR, we fused mCherry at the C terminus of PHR through a linker, as shown in Fig. 1A. mCherry has single excitation and emission peaks at 587 nm and 610 nm, respectively, and is not known to respond to nucleotides. Since this novel sensor protein resulted from PHR fusion with mCherry, we named it PHR-mCherry (Fig. 1A).

Since PHR-mCherry is a new sensor, we overexpressed the sensor in Escherichia coli and purified it to analyze whether it responds to ATP and ADP. As expected, spectral analysis using a fluorimeter revealed that PHR-mCherry possesses three excitation peaks. Two of the excitation peaks (Emi_max_, ∼525 nm) at 420 nm and 500 nm arise due to PHR, and one excitation peak (Emi_max_, 610 nm) is due to mCherry (Fig. 1B). It also possesses two emission peaks: one emission peak (Ex_max_, 500 nm) at 525 nm for PHR and another at 610 nm for mCherry (Fig. 1C). Importantly, incubation of this sensor with ATP resulted in an increase in the excitation peak at 500 nm, while incubation with ADP led to a decreased excitation peak at 500 nm (Fig. 1D). As expected, incubation of PHR-mCherry with ATP or ADP does not affect the fluorescence spectra of mCherry (Fig. 1D and E). Relevantly, the ratio of excitation of PHR at 500 nm and mCherry at 587 nm (500/587 ratio) could be effectively utilized for the measurement of ATP/ADP (Fig. 1F). Next, we analyzed whether other nucleotides such as CTP, UTP, GTP, AMP, and cAMP modulate PHR-mCherry. We did not observe any considerable effect of CTP, UTP, GTP, AMP, and cAMP on PHR-mCherry (Fig. S2A to C). We also analyzed the dynamic range of PHR-mCherry and observed that it could sense ATP within the range of 0.1 to 5 μM ATP (Fig. S3A). It was further seen that the sensor could sense ADP within the range of 0.1 to 2 μM ADP (Fig. S3B). We also analyzed if an increase in the sensor probe concentration affects the probe’s ratiometric behavior (500/587 ratio). Importantly, we observed that the increased protein concentration does not affect the 500/587 ratio (Fig. S3C). Finally, we analyzed the ability of the new sensor to respond to a range of ATP/ADP levels. Toward this, the ADP level was kept constant, and the ATP level was increased progressively. It was observed that similar to PHR, PHR-mCherry responded to ATP/ADP linearly within the range of 0.5 to 15 μM (Fig. S3D). Thus, the dynamic range of PHR-mCherry is similar to that of PHR (24, 25). These observations suggest that PHR-mCherry could work in mycobacterial cells to measure ATP/ADP, wherein the PHR domain will act as a sensory module, while mCherry is suitable for normalization.

10.1128/mBio.01088-21.2 FIG S2 Nucleotides other than ATP do not affect the spectral properties of purified PHR-mCherry. (A and B) A total of 10 μM PHR-mCherry protein was independently treated with 25 μM each CTP, UTP, GTP, cAMP, and AMP, and the excitation spectra (A) and emission spectra (B) were recorded using a fluorimeter. (C) Bar graph representing the fluorescence ratio at 500/587 nm of PHR-mCherry proteins in the control along with the CTP-, UTP-, GTP-, cAMP-, and AMP-treated sensor proteins. Data are expressed as means ± SEM from three independent experiments. Statistical significance was determined using a one-tailed unpaired *t* test. Download FIG S2, TIF file, 0.8 MB. Copyright © 2021 Akela and Kumar. 2021 Akela and Kumar. https://creativecommons.org/licenses/by/4.0/ This content is distributed under the terms of the Creative Commons Attribution 4.0 International license.

10.1128/mBio.01088-21.3 FIG S3 Dynamic range of PHR-mCherry. (A and B) A total of 10 μM PHR-mCherry protein was independently treated with various concentrations (0.1 to 25 μM) of ATP (A) and ADP (B), and the excitation peaks were recorded at 500 nm and 587 nm, keeping emission peaks fixed at 525 nm and 610 nm, respectively, using a fluorimeter. The 500/587 ratios of proteins in the presence of different concentrations of ATP and ADP were plotted as points and connecting lines using GraphPad Prism software. (C) Excitation peaks at 500 nm and 587 nm of proteins were taken with increasing concentrations of protein (2.5 μM to 15 μM), keeping ATP constant. The 500/587 ratios of protein were plotted as a column bar graph using GraphPad Prism software. (D) The dynamic range of PHR-mCherry was determined by exposing PHR-mCherry to different ATP/ADP ratios followed by measurement of fluorescence. A range of ATP/ADP concentrations was acquired using increasing concentrations of ATP and keeping ADP (10 μM) constant. The excitation peaks were recorded at 500 nm and 587 nm, keeping emission peaks fixed at 525 nm and 610 nm, respectively, using a fluorimeter. The 500/587 ratios of protein were plotted as points and a connecting line graph. The data presented here are representative of results from three experiments performed in technical triplicate. Each dot represents the mean ± SEM from the technical triplicate. **, *P* < 0.01. Download FIG S3, TIF file, 0.9 MB. Copyright © 2021 Akela and Kumar. 2021 Akela and Kumar. https://creativecommons.org/licenses/by/4.0/ This content is distributed under the terms of the Creative Commons Attribution 4.0 International license.

### Validation of PHR-mCherry in mycobacterial cells.

**FIG 2:**
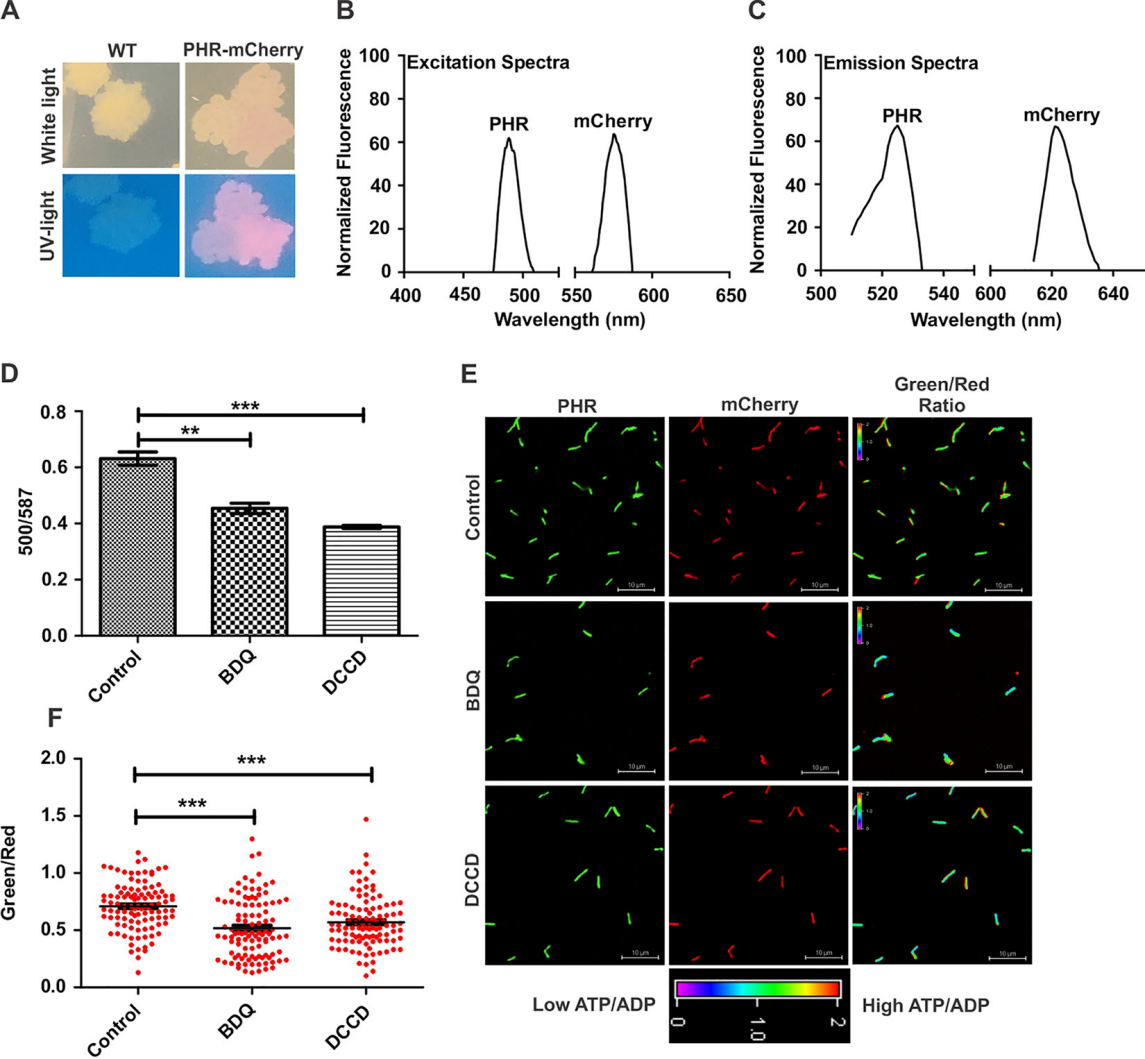
Validation of the ATP/ADP reporter strain of Mtb. (A) The Mtb reporter strain overexpressing PHR-mCherry was cultured on a 7H10 agar plate, and pictures of the colonies were taken in the presence of normal light and UV light. Colonies of Mtb overexpressing PHR-mCherry are pink. The pink color due to the overexpression of the ATP/ADP reporter was better visualized in UV light. WT, wild type. (B and C) Excitation spectra (B) and emission spectra (C) of the Mtb reporter strain in the log phase of growth. (D) A log-phase culture of the reporter strain of Mtb was independently treated with 0.35 μM BDQ and 100 μM DCCD for 6 h, and the 500/587 ratio was determined using a fluorimeter. The 500/587 ratio corresponds to the cellular ATP/ADP levels. (E and F) The samples described above were also subjected to confocal microscopy, followed by analysis using NIS-elements. Confocal images were captured using lasers of 488 nm and 561 nm by CLSM. These images were processed to generate images depicting pseudocolor fluorescence to present the individual cells’ ATP/ADP levels. The color scale for the ratio values indicates high and low ATP/ADP ratios. The scatterplot of the ATP/ADP ratios in bacteria was plotted using GraphPad Prism software. Each dot represents ATP/ADP levels in an individual bacterium. The color bar represents green/red ratios. The data are expressed as the means ± SEM from three independent experiments performed in triplicates (*n* = 100 bacteria). A two-tailed unpaired *t* test was performed to determine the statistical significance of data. **, *P* < 0.01; ***, *P* < 0.001.

To test whether the sensor could be useful for measuring ATP/ADP in mycobacterial cells, codon-optimized PHR-mCherry was cloned into the integrating shuttle vector pMV761 (33). pMV761 is derived from pMV361; this vector utilizes integrase from mycobacteriophage L5, and its integration site is well defined (34). Constitutive overexpression in this vector is derived through the *hsp60* promoter. pMV761-PHR-mCherry was transformed into slow-growing Mtb and fast-growing Msmeg. Transformed colonies of Mtb and Msmeg appeared pink on 7H10 agar in visible light due to the presence of mCherry. The same bacterial colonies appeared green in UV light due to YFP (Fig. 2A) and were subsequently used for monitoring mycobacterial ATP/ADP levels. To further confirm the expression of the ATP/ADP reporter (PHR-mCherry) strain of Mtb, we acquired fluorescence spectra. As expected, we observed an excitation peak at 500 nm and one emission peak at 525 nm due to PHR (Fig. 2B and C). Additionally, excitation/emission peaks of mCherry were observed (Fig. 2B and C). The same excitation and emission profiles were also observed in the reporter strain of Msmeg (Fig. S4A and B). Notably, the overexpression of PHR-mCherry did not affect mycobacterial growth (Fig. S5A and B). To determine whether PHR-mCherry responds to cellular ATP/ADP levels, we exposed Mtb cells overexpressing PHR-mCherry to the ATP synthase inhibitors BDQ and DCCD. BDQ- and DCCD-mediated ATP reduction was also confirmed by measuring ATP levels in crude cell extracts using the luciferase assay (Fig. S6). A considerable decrease in the 500/587 ratio was observed in the newly constructed reporter strain upon exposure to BDQ and DCCD, suggesting that the sensor PHR-mCherry is responsive to cellular ATP/ADP (Fig. 2D). Since these were fluorometric analyses that reflect the population’s ensemble average, we utilized confocal laser scanning microscopy (CLSM) for measuring ATP/ADP at the single-cell level. CLSM confirmed the functionality of the newly developed reporter strain of Mtb and divulged the ATP/ADP levels to the single bacterial cell (Fig. 2E and F). CLSM analysis also revealed a broad heterogeneity in ATP/ADP in the population of bacteria in the same culture (Fig. 2E and F) that has not been considered in previous studies. Similar observations were made in Msmeg (Fig. S7A to C). These observations thus suggested that the reporter strains of Mycobacterium are functional.

10.1128/mBio.01088-21.4 FIG S4 Expression of the PHR-mCherry reporter strains of Msmeg. (A) The excitation spectra of PHR-mCherry were taken at Emi_max_ values of 525 nm and 610 nm, and the graph of excitation spectra was plotted as a connecting line using GraphPad Prism software. (B) The emission spectra of PHR-mCherry were taken at Ex_max_ values of 500 nm and 587 nm, and the emission spectra of PHR-mCherry were plotted as a connecting line. Download FIG S4, TIF file, 0.3 MB. Copyright © 2021 Akela and Kumar. 2021 Akela and Kumar. https://creativecommons.org/licenses/by/4.0/ This content is distributed under the terms of the Creative Commons Attribution 4.0 International license.

10.1128/mBio.01088-21.5 FIG S5 Overexpression of PHR-mCherry does not affect the growth of Msmeg (A) and Mtb (B). Growth curves of Msmeg (A) and Mtb (B) reporter strains (red dots) and the vector control (blue dots) are shown. Graphs were plotted using points and connecting lines using GraphPad Prism software. The data are means ± SEM and are representative of results from three independent experiments. Download FIG S5, TIF file, 0.5 MB. Copyright © 2021 Akela and Kumar. 2021 Akela and Kumar. https://creativecommons.org/licenses/by/4.0/ This content is distributed under the terms of the Creative Commons Attribution 4.0 International license.

10.1128/mBio.01088-21.6 FIG S6 Validation of BDQ- and DCCD-mediated depletion of ATP by a luciferase assay. The ATP/ADP reporter strain of Mtb was incubated with BDQ (0.35 μM) and DCCD (100 μM) for 3 h, and the cells were then lysed, followed by a luciferase assay. The bioluminescence intensities of control and treated samples were plotted as a column bar graph using GraphPad Prism software. The data are representative of results from three experiments in triplicate. Data are presented as means ± SEM. The significance of data was calculated by a one-tailed unpaired *t* test. ***, *P* < 0.001. Download FIG S6, TIF file, 0.2 MB. Copyright © 2021 Akela and Kumar. 2021 Akela and Kumar. https://creativecommons.org/licenses/by/4.0/ This content is distributed under the terms of the Creative Commons Attribution 4.0 International license.

10.1128/mBio.01088-21.7 FIG S7 Validation of the PHR-mCherry reporter strains of Msmeg. (A) The reporter strain of Msmeg was treated with BDQ (0.35 μM), and ATP/ADP levels were determined using CLSM. Confocal images were captured using 488-nm and 561-nm lasers after treatment of the reporter strain of Msmeg with BDQ for 3 h. The data were analyzed by NIS-elements, and the green/red ratios of bacteria were plotted as a scatterplot using GraphPad Prism software. The color scale bar indicates low and high ATP/ADP ratios. (B) ATP/ADP levels in the bacterial population depicted as scatterplots generated using GraphPad Prism software. Each dot represents the intracellular ratio of ATP/ADP of an individual bacterium. (C) A column bar graph was plotted to depict the ATP/ADP levels of the mycobacterial population (Msmeg), and the 500/587 ratio was obtained from fluorimetry analysis of 3 h samples. Data are means ± SEM and are representative of results from three independent biological experiments performed in technical replicates. The significance of data was calculated by a one-tailed unpaired *t* test. *, *P* < 0.05; ***, *P* < 0.001. Download FIG S7, TIF file, 0.9 MB. Copyright © 2021 Akela and Kumar. 2021 Akela and Kumar. https://creativecommons.org/licenses/by/4.0/ This content is distributed under the terms of the Creative Commons Attribution 4.0 International license.

To further confirm the probe’s suitability for measuring ATP/ADP levels, we exposed the PHR-mCherry-overexpressing Mtb cultures to arsenate V (AsnV). Interestingly, AsnV could be used as a substrate by ATP synthase, and thus, treatment of cells with arsenate V results in the formation of ADP-AsnV instead of ATP (35, 36). ADP-AsnV is a structural analogue of ATP, and many enzymes cannot differentiate between ADP-AsnV and ATP (36). Noticeably, we observed that treatment of Mtb with AsnV resulted in a significant depletion of ATP/ADP levels (Fig. S8A and B). Expectedly, the level of ATP/ADP depletion was similar to those with BDQ and DCCD (Fig. S8A and B). These findings suggest that the new probe is highly specific for ATP and does not sense ADP-AsnV. In summary, the above described observations indicate that PHR-mCherry is a highly specific probe that could monitor the metabolic state of mycobacteria.

10.1128/mBio.01088-21.8 FIG S8 ATP/ADP levels in Mtb in the presence of arsenate, BDQ, and DCCD. The reporter strain of Mtb was incubated with arsenate (5 mM), BDQ (0.35 μM), and DCCD (100 μM) for 3 h, followed by confocal microscopy and data analysis. (A) Confocal images of Mtb were captured using 488-nm and 561-nm lasers, and pseudocolors were also captured (green/red). (B) Green/red ratios of Mtb plotted as a scatterplot using GraphPad Prism software. The color scale bar indicates low and high ATP/ADP ratios (violet to red). Data are means ± SEM and are representative of results from three independent biological experiments performed in technical replicates. The significance of data was calculated by a one-tailed unpaired *t* test. ***, *P* < 0.001. Download FIG S8, TIF file, 0.8 MB. Copyright © 2021 Akela and Kumar. 2021 Akela and Kumar. https://creativecommons.org/licenses/by/4.0/ This content is distributed under the terms of the Creative Commons Attribution 4.0 International license.

### Effects of antimycobacterial drugs on bioenergetics of Mtb *in vitro*.

**FIG 3:**
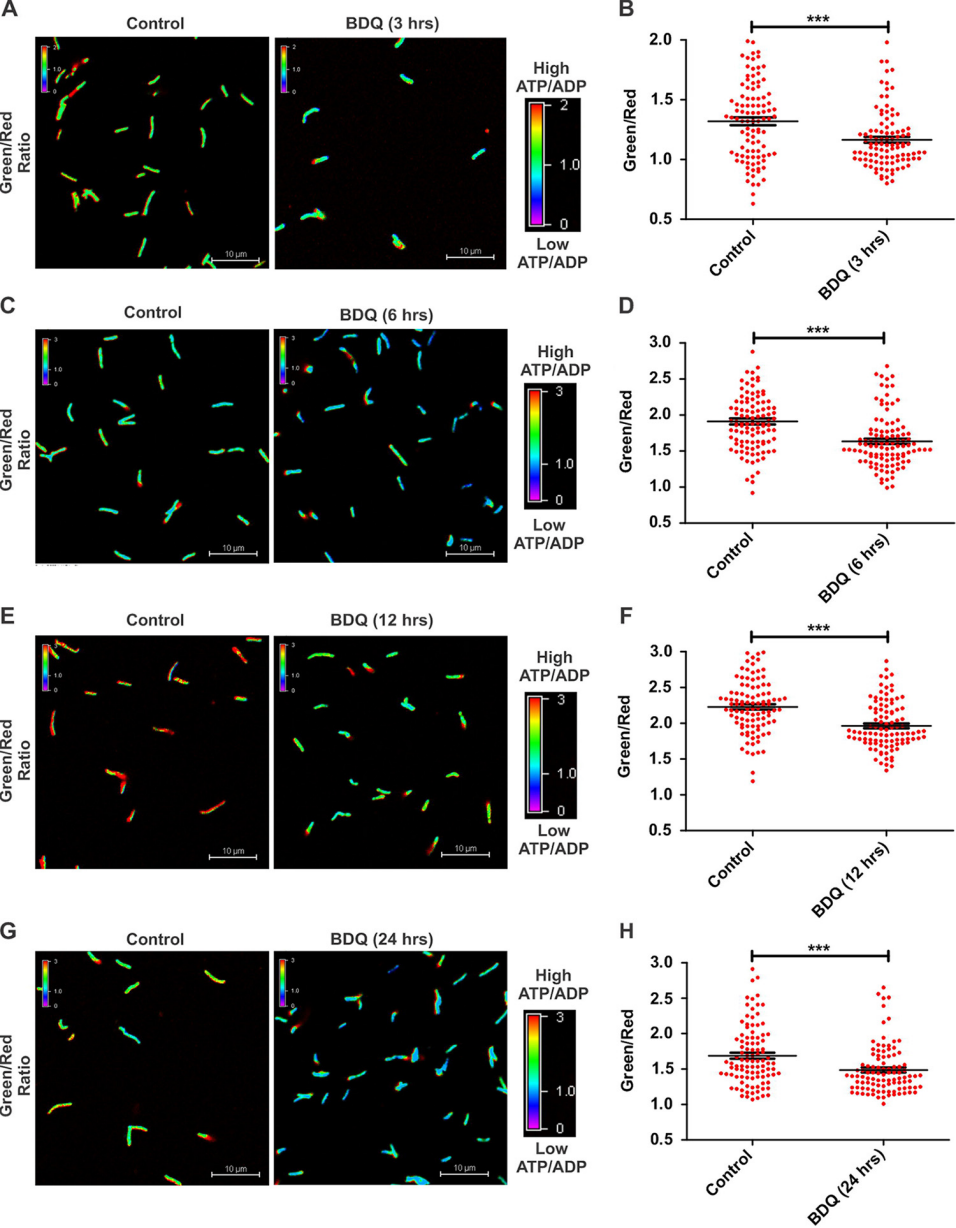
Dynamics of BDQ-mediated ATP depletion in Mtb cells. A log-phase culture of the reporter strain of Mtb was treated with 0.35 μM BDQ for 3 h (A and B), 6 h (C and D), 12 h (E and F), and 24 h (G and H) and subjected to confocal microscopy followed by analysis using NIS-elements. Confocal images were captured using 488-nm and 561-nm lasers and processed by CLSM. Panel A depicts representative confocal images of the Mtb reporter strain exposed to BDQ for 3 h. The ATP/ADP (green/red) ratio was calculated from captured images and expressed as pseudocolored fluorescence ratiometric images (green/red) representing intracellular ATP/ADP corresponding to the scale bar. ATP/ADP levels in the bacterial population are depicted as scatterplots generated using GraphPad Prism software. Each dot in panels B, D, F, and H represents the intracellular ratio of ATP/ADP of an individual bacterium. Data are presented as means ± SEM and are representative of results from three independent experiments performed in triplicates (*n* = 100 bacteria). Statistical significance was determined using a two-tailed unpaired *t* test. ***, *P* < 0.001.

Next, we used this reporter strain to examine the temporal effect of BDQ on ATP/ADP of Mtb. Toward this, we exposed Mtb cells to BDQ and analyzed them for ATP/ADP levels at 1 h, 2 h, 3 h, 6 h, 12 h, and 24 h using CLSM. After about 3 h of BDQ treatment, the ATP/ADP levels of the population as a whole became significantly lower than those of the control population (Fig. 3A and B). After this time point, the ATP/ADP levels of Mtb remained lower at least until 24 h (Fig. 3C to H). Interestingly, some cells maintained higher levels of ATP/ADP at all time points. These observations point toward the inherent heterogeneity in a bacterial population.

**FIG 4:**
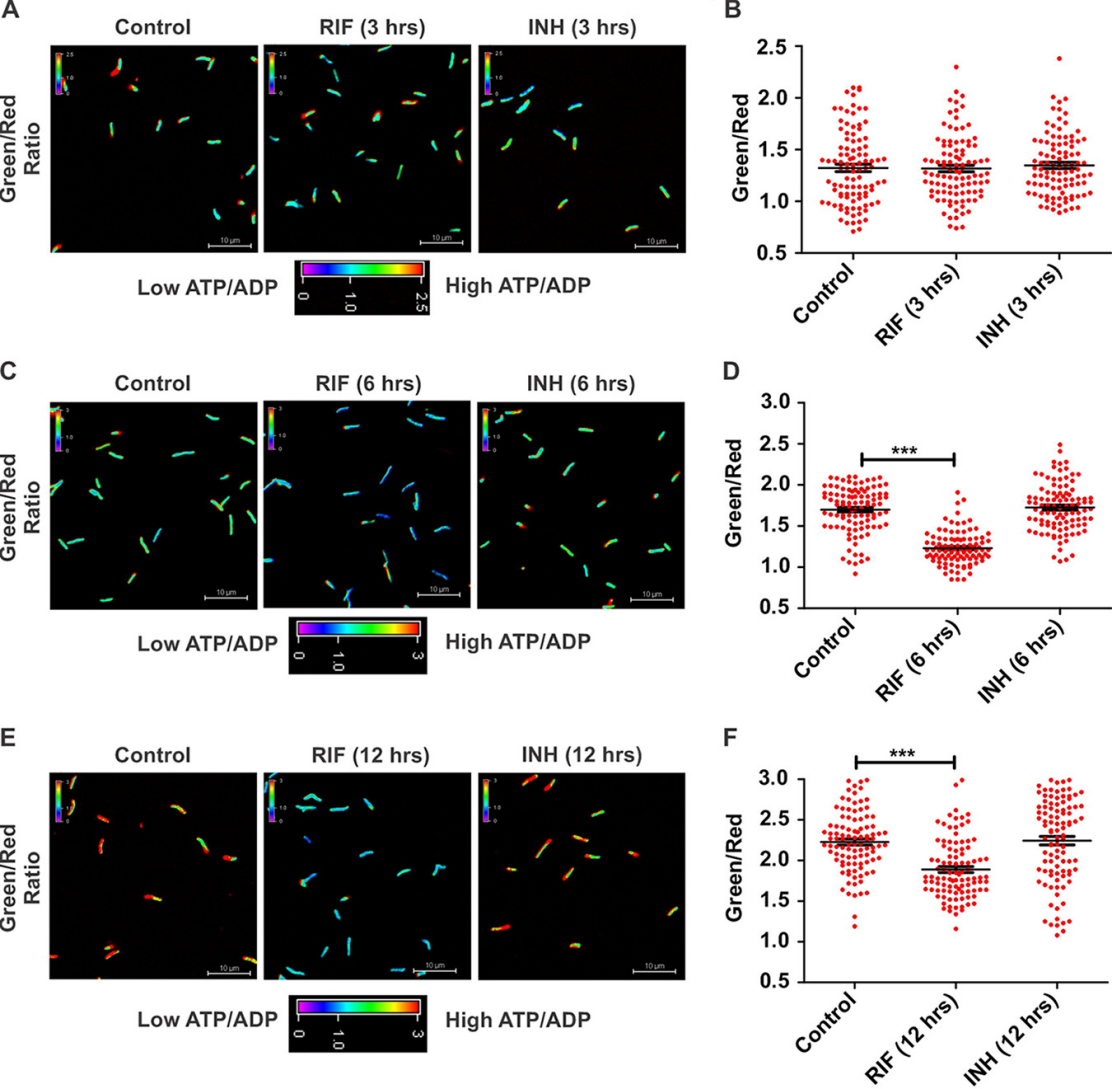
Effects of isoniazid and rifampicin on ATP/ADP levels in Mtb. A log-phase culture of the ATP/ADP reporter strain of Mtb was treated with RIF and INH. The samples were fixed at 3 h (A and B), 6 h (C and D), and 12 h (E and F), followed by confocal microscopy. Confocal images were captured using 488-nm and 561-nm lasers. These images were used for the calculation of green/red ratios and are depicted as pseudocolored fluorescence ratiometric images. Scatterplots of single-cell ratios in panels B, D, and F were drawn using GraphPad Prism software. Each dot in panels B, D, and F represents the intracellular ratio of ATP/ADP of an individual bacterium. Data are presented as means ±SEM and are representative of results from three independent experiments performed in triplicates (*n* = 100 bacteria). Statistical significance was determined using a two-tailed unpaired *t* test. ***, *P* < 0.001.

Previous studies suggest a critical connection between antimycobacterial drugs and the metabolic state of Mtb (37–39). ATP homeostasis is crucial for the survival of mycobacteria. Thus, we analyzed whether the frontline antimycobacterial drugs INH and RIF affect the metabolic state of Mtb using the ATP/ADP reporter strain of Mtb. Mtb cultures were independently exposed to INH and RIF for 3 h, 6 h, and 12 h, and the ATP/ADP levels of the bacilli were accessed by confocal microscopy. We chose these time points to avoid the effects of drug-mediated killing on metabolism. No change in the ATP/ADP levels was observed after 3 h of treatment with both drugs (Fig. 4A and B). Interestingly, we observed that RIF exposure leads to a consistent and significant decrease in ATP/ADP levels after 6 h (Fig. 4C and D), and this effect on metabolism can be observed at 12 h as well (Fig. 4E and F). On the contrary, the cell wall synthesis inhibitor INH could not alter ATP/ADP of Mtb (Fig. 4C to F). It is worth noting that the ATP synthase inhibitor was able to change the ATP/ADP levels quickly, but the transcription inhibitor RIF needs a longer time to deplete the ATP/ADP levels (Fig. 3 and 4). These results are interesting, although the molecular mechanism underlying the difference remains unknown.

### Phagocytosis leads to depletion of ATP/ADP levels in Mycobacterium.

**FIG 5:**
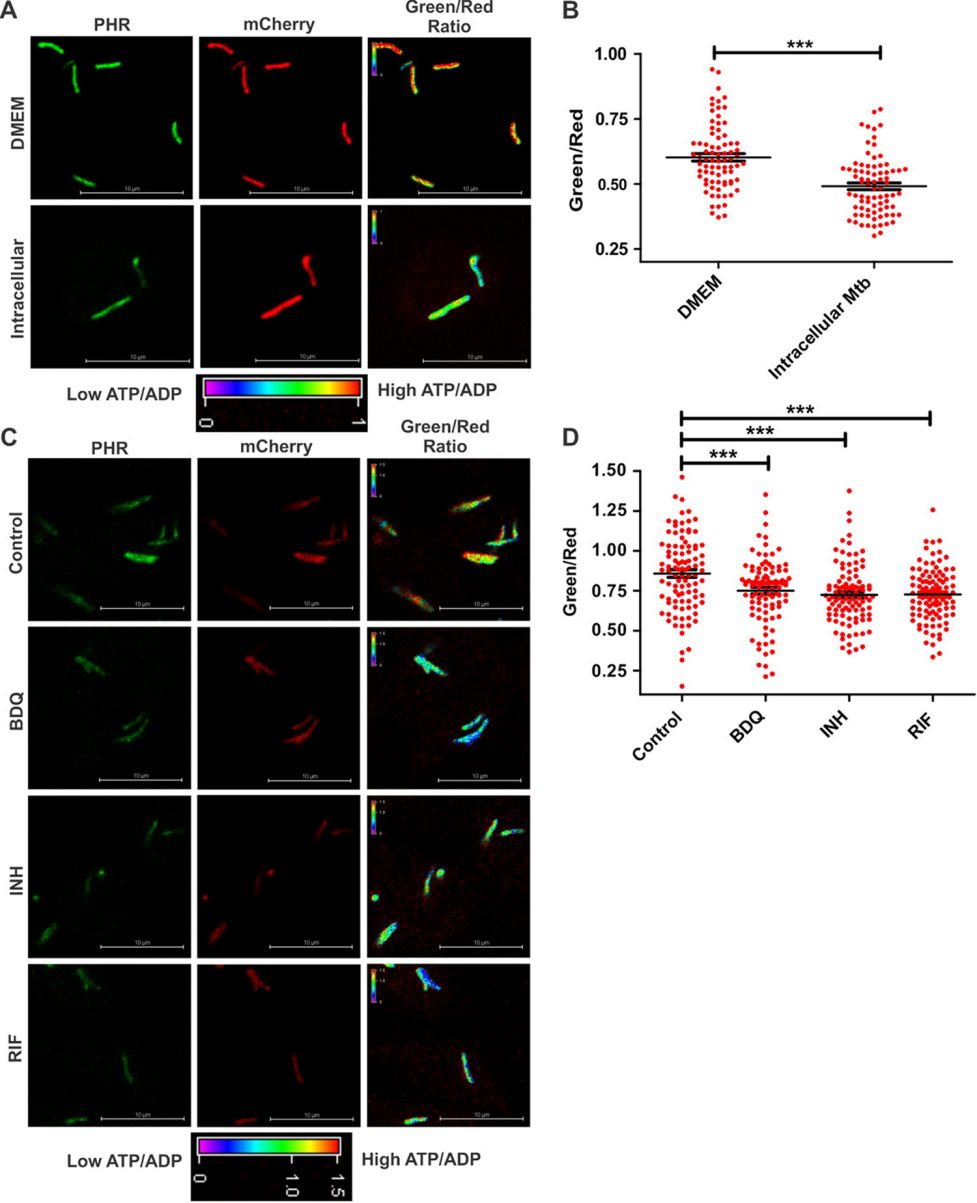
ATP/ADP levels in intracellular Mtb. (A and B) ATP/ADP ratios of Mtb cells residing inside macrophages (RAW 264.7) for 3 h or extracellular bacilli in DMEM. (A) Images of extracellular (DMEM) and intracellular Mtb. (B) Images acquired using confocal microscopy and plotted as a scatterplot using GraphPad Prism software. (C) RAW 264.7 macrophages were infected with the reporter strain of Mtb and then independently treated with BDQ, RIF, and INH (5× MIC each) for 6 h. Confocal images were acquired using 488-nm and 561-nm lasers. The pseudocolored fluorescence images depict the ATP/ADP levels of the intracellular bacteria. (D) Scatterplots of the ATP/ADP ratios. Data in panels B and D are presented as means ± SEM and are representative of results from three independent biological experiments performed in technical triplicates (*n* = 100 bacteria). Each dot in panels B and D represents the intracellular ratio of ATP/ADP of an individual bacterium. Statistical significance was determined using a two-tailed unpaired *t* test. ***, *P* < 0.001.

Next, we utilized the new reporter strain of Mtb to monitor the effect of phagocytosis on the ATP/ADP level of the bacterium during infection. For this purpose, we infected RAW 264.7 cells with the Mtb reporter strain overexpressing PHR-mCherry at a multiplicity of infection (MOI) of 1:10 and then measured the ATP/ADP ratio in intracellular Mtb and compared it with the ratio in extracellular Mtb in Dulbecco’s modified Eagle’s medium (DMEM) as a control. We observed significantly lower ATP/ADP levels in intracellular Mtb than in extracellular Mtb (Fig. 5A and B). These observations were also validated using the traditional method of ATP/ADP estimation (Fig. S9). The reason for the decrease in ATP levels of intracellular Mtb could be the environmental stress and nutrient starvation faced by Mtb inside infected macrophages.

10.1128/mBio.01088-21.9 FIG S9 Measurement of ATP/ADP levels of intracellular Mtb and extracellular Mtb using a conventional method. RAW 264.7 macrophages were infected with Mtb at an MOI of 1:10. Mtb cells that did not infect macrophages were recovered from the medium, while intracellular Mtb cells were isolated at 3 h postinfection by lysis of macrophages. Mtb cells were lysed by bead beating, and the ATP/ADP level of the bacterial population was measured using an ATP/ADP assay kit (Sigma-Aldrich). The bar graph was plotted by using GraphPad Prism software. Data are presented as means ± SEM and are representative of results from three independent experiments performed in triplicates (*n* = 3). Statistical significance was determined using a two-tailed unpaired *t* test. ***, *P* < 0.001. Download FIG S9, TIF file, 0.3 MB. Copyright © 2021 Akela and Kumar. 2021 Akela and Kumar. https://creativecommons.org/licenses/by/4.0/ This content is distributed under the terms of the Creative Commons Attribution 4.0 International license.

### Effects of antimycobacterial drugs on ATP/ADP levels of intracellular Mtb.

Several studies have reported that antimycobacterial drugs induce metabolic stress in intracellular Mtb (40–43). Subsequently, we studied the effect of antimycobacterial drugs on the ATP/ADP homeostasis of intracellular Mtb. For this purpose, we infected the RAW 264.7 cells with an ATP/ADP reporter strain of Mtb. Infected macrophages were treated with BDQ, INH, and RIF for 6 h, and ATP/ADP of intracellular Mtb was then determined using CLSM. As expected, we observed that BDQ induced a significant depletion of ATP/ADP levels in intracellular Mtb (Fig. 5C and D). Surprisingly, INH and RIF were able to cause ATP/ADP level depletion, similarly to BDQ in intracellular Mtb (Fig. 5C and D). These observations were in contrast to *in vitro* observations and suggest that inside macrophages, Mtb is faced with metabolic stress, and inhibition of other vital processes such as transcription or cell wall synthesis leads to a further downshift in metabolism. Alternatively, lowering ATP/ADP levels could be one of the mycobacterial ways of intracellular Mycobacterium to respond to a plethora of different types of stresses, including antibiotic stress.

### Activation of macrophages by interferon gamma leads to decreased ATP/ADP levels in intracellular Mtb.

**FIG 6:**
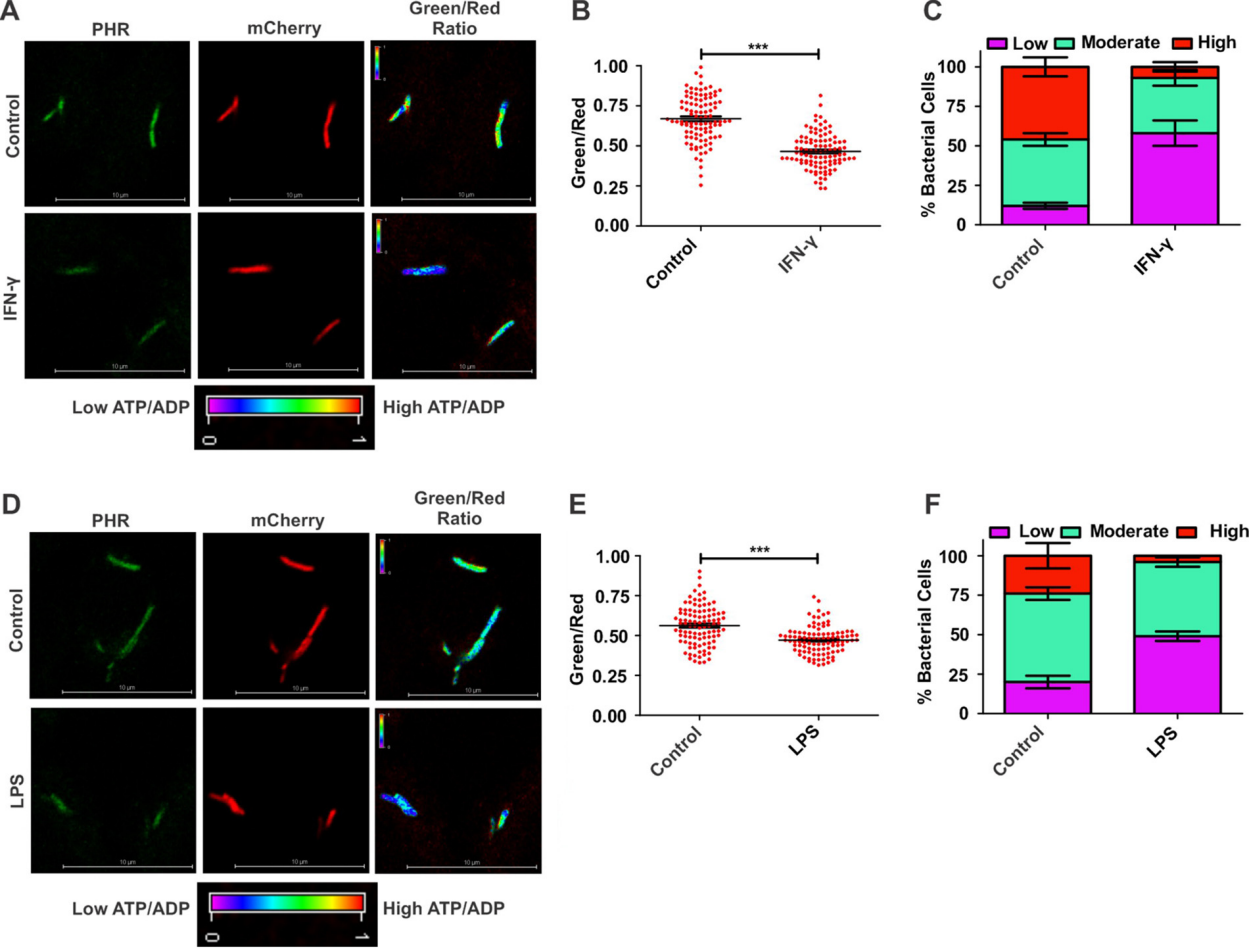
Macrophage activation leads to a decrease in ATP/ADP levels in intracellular Mtb. RAW 264.7 macrophages were activated with IFN-γ or LPS, and the cells were then infected with Mtb at a multiplicity of infection (MOI) of 1:10 for 3 h. At 3 h postinfection, the cells were fixed and analyzed using confocal microscopy. (A) Confocal image of IFN-γ-activated RAW 264.7 cells infected with the ATP/ADP reporter strain of Mtb. (B) Scatterplot of ATP/ADP levels of mycobacteria inside IFN-γ-activated or naive macrophages. The green/red ratios were calculated using NIS-elements software. (C) Stacked bar graph representing intracellular Mtb distributions into fractions with high, moderate, and low ATP/ADP levels. (D) Confocal images of intracellular Mtb cells residing inside LPS-activated cells. (E) Scatterplot of ATP/ADP ratios of intracellular bacteria at the single-cell level in LPS-activated macrophages. (F) Stacked bar graph showing the distribution of Mtb in LPS-activated macrophages into fractions with high, moderate, and low ATP/ADP levels. The data shown in panels B, C, E, and F are presented as means ± SEM and are representative of results from three independent biological experiments performed in technical triplicate (*n* = 100 bacteria). Statistical significance was determined using a two-tailed, unpaired *t* test. ***, *P* < 0.001.

Interferon gamma (IFN-γ) and bacterial lipopolysaccharides (LPS) are known to activate macrophages (44, 45), which leads to the production of reactive oxygen species (ROS) and reactive nitrogen species (RNS) and facilitates phagosome maturation. ROS and RNS inhibit the growth of intracellular Mtb in activated macrophages (46). To understand the effect of macrophage activation on the bioenergetics of intracellular Mtb, we activated the macrophages using IFN-γ and infected them with the reporter strain. ATP/ADP levels of Mtb residing in IFN-γ-activated macrophages and resting macrophages were measured using confocal microscopy. We found that the ATP/ADP ratio of Mtb residing in activated macrophages (RAW 264.7 cells) was lower than that in naive macrophages (Fig. 6A and B). To better understand the metabolic heterogeneity in the metabolic state, we divided the bacterial population among three fractions according to their metabolic state: (i) bacteria having high ATP/ADP levels, (ii) bacteria with moderate ATP/ADP levels, and (iii) bacteria with low levels of ATP/ADP. We found that a significant fraction of the bacterial populations residing inside naive macrophages has a high ATP/ADP ratio (Fig. 6C). Interestingly, IFN-γ treatment leads to a significant reduction in this population and leads to an increase in the fraction with low ATP/ADP levels (Fig. 6C). A similar effect on the metabolic state of Mtb was seen upon treatment of RAW 264.7 cells with LPS (Fig. 6D to F).

### Localization of Mtb in different subvacuolar compartments dictates the bioenergetic heterogeneity of intracellular Mtb.

**FIG 7:**
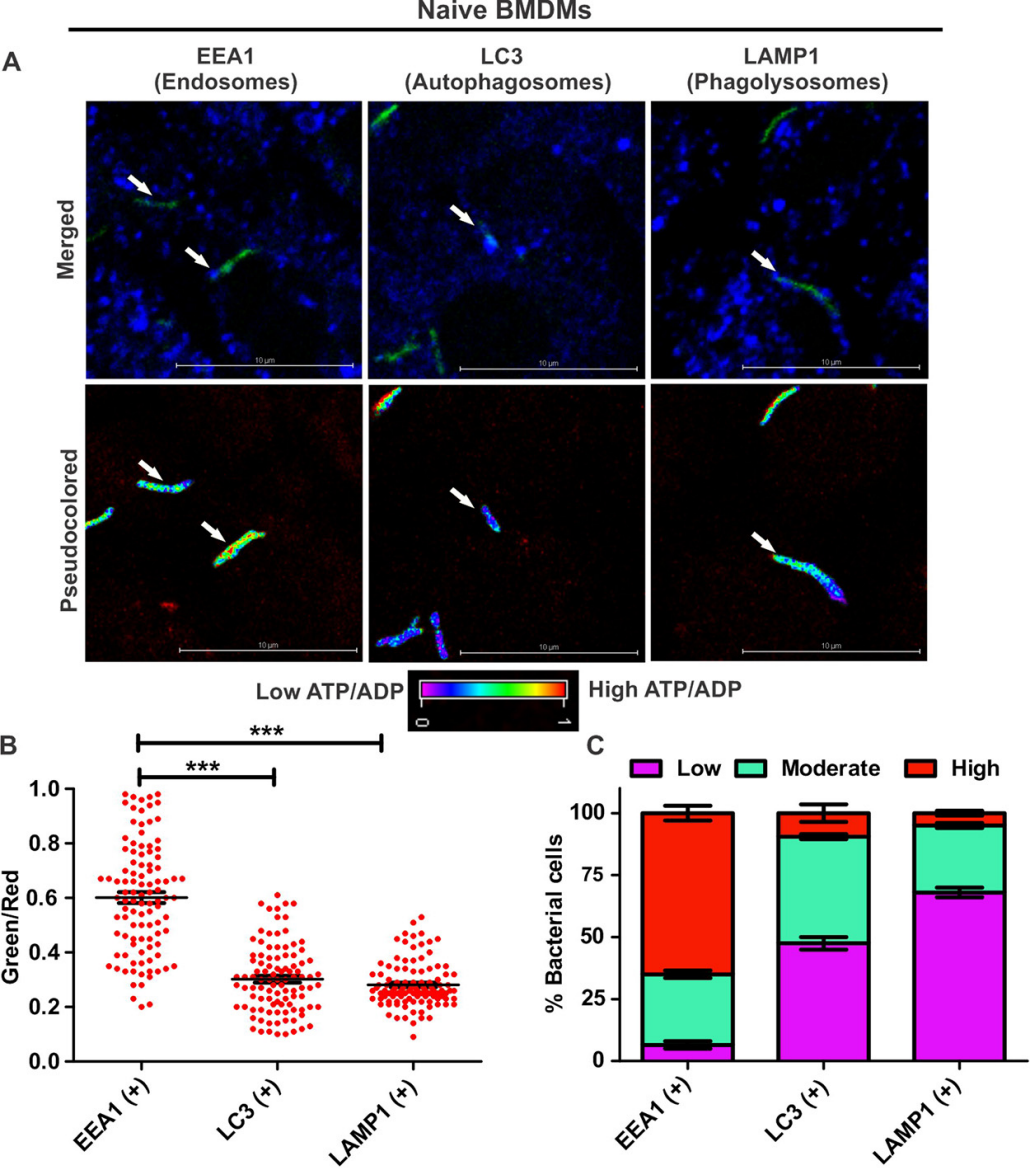
The subcellular localization dictates the ATP/ADP levels of intracellular Mtb residing in naive macrophages. BMDMs were infected with the ATP/ADP reporter strain of Mtb (MOI = 1:10) for 3 h. The subcellular localization of intracellular Mtb was determined by staining with antibodies against EEA1, LAMP1, and LC3, followed by confocal microscopy to mark the early endosomes, phagolysosomes, and autophagosomes, respectively. (A) Representative confocal images showing the colocalization of subvacuolar markers (blue) with bacteria (green) and their respective pseudocolored ratiometric images of colocalized bacteria to depict the ATP/ADP levels in that particular bacterium. (B) Scatterplots representing the ATP/ADP (green/red) levels of the bacteria residing inside the early endosomes/phagosomes, phagolysosomes, and autophagosomes. Each dot represents the intracellular ratio of ATP/ADP of an individual bacterium. (C) Stacked bar plots representing the distributions of populations with low, moderate, and high ATP/ADP ratios of bacteria colocalizing with EEA1, LC3, and LAMP1. Data were plotted using GraphPad Prism software after the analysis of captured images. Data shown are presented as means ± SEM and are illustrative of results from three independent experiments performed in triplicates (*n* = 100 bacteria). Statistical significance was determined by using a two-tailed unpaired *t* test. ***, *P* < 0.001.

Mtb resides inside macrophages in several subcellular niches such as early phagosomes, autophagosomes, and phagolysosomes. However, the metabolic state of Mtb in different subcellular niches is not defined. We hypothesized that Mtb cells residing in separate subcellular compartments might possess different ATP/ADP levels, which may alter their physiology. To test this hypothesis, we infected bone marrow-derived macrophages (BMDMs) with ATP/ADP reporter strains of Mtb for 3 h. We measured the ATP/ADP ratio of bacteria residing in different subvacuolar compartments using CLSM. We specifically choose BMDMs for these experiments as they closely resemble macrophages encountered during infection of mammalian hosts (47, 48). Furthermore, BMDMs are larger than RAW 264.7 macrophages and thus are more conducive to identifying a particular subvacuolar compartment. Here, we used EEA1 for locating the Mtb bacteria residing in early endosomes/phagosomes (49). LAMP1 (lysosome-associated membrane protein 1) was used as a marker for late endosomes/lysosomes (referred to here as phagolysosomes) (50), and LC3 (microtubule-associated protein 1A/1B light chain 3) (51) was used for tracking Mtb residing inside autophagosomes. Interestingly, we observed that the bacilli residing in phagosomes possess significantly higher ATP/ADP levels than the bacilli residing inside autophagosomes and phagolysosomes at the ensemble population levels (Fig. 7A and B). We binned the population into three categories as described above. It was evident that a significant fraction of bacilli residing inside early endosomes/phagosomes has high levels of ATP (Fig. 7C). The fraction with high ATP/ADP levels is significantly decreased in the population of bacilli residing inside autophagosomes and phagolysosomes (Fig. 7C). Notably, a large fraction of the bacilli residing inside autophagosomes and phagolysosomes possess moderate or low ATP/ADP levels (Fig. 7C). These observations indicate differences in the bioenergetic states of Mtb cells residing inside different subvacuolar compartments.

### Activation of macrophages impacts the bioenergetics state of bacilli residing inside autophagosomes and phagolysosomes.

**FIG 8:**
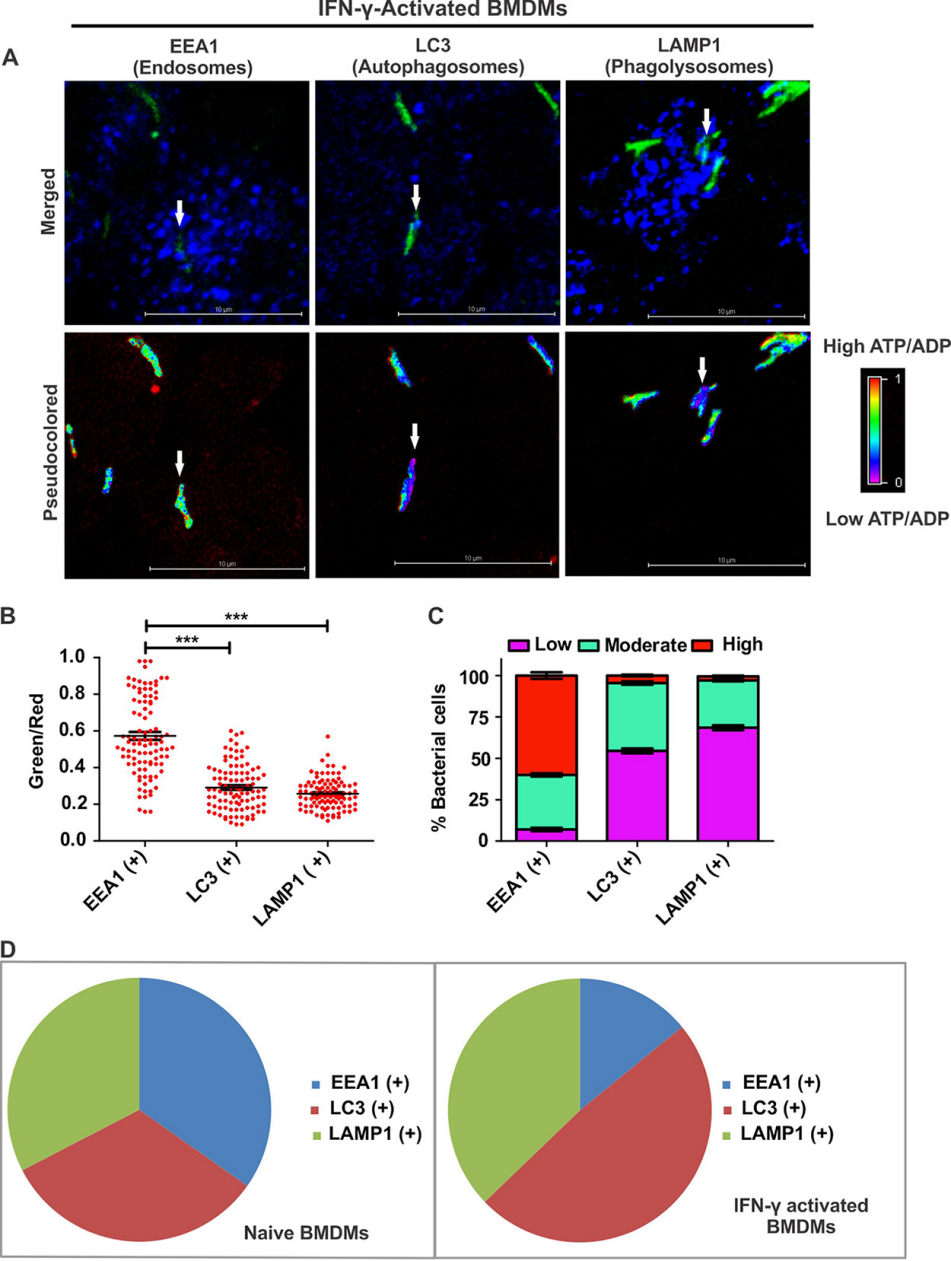
Activation of macrophages modulates ATP/ADP levels of intracellular Mtb through the trafficking of bacilli into phagolysosomes and autophagosomes. IFN-γ-activated BMDMs were infected with the ATP/ADP reporter strain of Mtb for 3 h. The subvacuolar localization of intracellular bacilli was defined using staining with antibodies specific for EEA1, LAMP1, and LC3, followed by confocal microscopy to mark the early endosomes, phagolysosomes, and autophagosomes, respectively. (A) Representative confocal images depicting the colocalization of subvacuolar markers (blue) with bacteria (green) and their respective pseudocolored fluorescence ratiometric images of colocalized bacteria to depict the bacterial ATP/ADP levels. (B) Scatterplots representing the ATP/ADP (green/red) levels of the bacteria residing inside the early endosomes/phagosomes, phagolysosomes, and autophagosomes. Every dot represents the intracellular levels of ATP/ADP in a bacterium. (C) Stacked bar plots representing the distributions of the bacterial population having low, moderate, and high ATP/ADP levels and colocalizing with EEA1, LC3, and LAMP1. Data shown are presented as means ± SEM and are representative of results from three independent experiments performed in triplicates (*n* = 100 bacteria). Statistical significance was determined by using a two-tailed unpaired *t* test. ***, *P* < 0.001. (D) Distribution of intracellular Mtb residing in phagosomes, phagolysosomes, and autophagosomes of naive and activated BMDM cells. The pie charts were plotted using GraphPad Prism software after the analysis of captured images.

Consequently, we analyzed the metabolic state of the bacilli residing in separate compartments of IFN-γ-activated macrophages. Interestingly, we observed that the Mtb cells residing in early phagosomes had higher ATP/ADP levels than bacilli residing in autophagosomes and phagolysosomes (Fig. 8A and B). Binning-based analysis suggested that the fractions of Mtb cells possessing low and moderate ATP levels were higher in autophagosomes and phagolysosomes (Fig. 8C). These observations were similar to those for naive macrophages. We also compared the metabolic states of the mycobacteria residing inside early phagosomes, autophagosomes, and phagolysosomes between the activated and naive macrophages using data from the above-described experiments. We observed that the ensemble population levels in the metabolic state of Mtb residing in the early phagosomes (Fig. S10A and B) and autophagosomes (Fig. S10C and D) in naive and activated macrophages are similar. However, we observed significantly lower ATP/ADP levels in the Mtb cells residing inside the phagolysosomes of the activated macrophages than in the naive macrophages (Fig. S10E and F). Further data analysis using ATP/ADP level-based binning suggests that the proportion of Mtb cells with lower ATP/ADP levels increases inside Mtb cells residing in the autophagosomes/phagolysosomes (Fig. S10D and F). We also analyzed the distribution of Mtb cells residing within these subcellular compartments in naive and activated macrophages. We observed that the intracellular bacteria are homogeneously spread in these three compartments in naive macrophages. However, interferon gamma-mediated activation of macrophages leads to the maturation of Mtb-containing phagosomes into autophagosomes and phagolysosomes (Fig. 8D), wherein Mtb faces metabolic stress.

10.1128/mBio.01088-21.10 FIG S10 ATP/ADP levels in Mtb residing in different subvacuolar compartments of naive and activated BMDM cells. (A, C, and E) Scatterplots of ATP/ADP levels in bacteria residing inside the EEA1-positive compartment (A), the LC3-positive compartment (C), and the LAMP1-positive compartment (E). (B, D, and F) Stacked bar graphs of the metabolic state of bacteria residing in the EEA1-positive compartment (B), the LC3-positive compartment (D), and the LAMP1-positive compartment (F) were plotted by calculating the fluorescence ratios of captured confocal images. The bacterial population was grouped into three populations: low, moderate, and high ATP/ADP ratios. Data are presented as means ± SEM and are representative of results from three independent experiments performed in triplicates (*n* = 100 bacteria). Statistical significance was determined by using a two-tailed unpaired *t* test. *, *P* < 0.05. Download FIG S10, TIF file, 1.2 MB. Copyright © 2021 Akela and Kumar. 2021 Akela and Kumar. https://creativecommons.org/licenses/by/4.0/ This content is distributed under the terms of the Creative Commons Attribution 4.0 International license.

## DISCUSSION

ATP/ADP plays a critical role in regulating the metabolic flux of bacterial cells, including Mtb. Currently used methods are unable to provide spatiotemporal details of changes in ATP/ADP levels and thus are unsuitable for monitoring the metabolism of Mtb during infection. In this study, we have reengineered PHR into a novel sensor named PHR-mCherry that is capable of monitoring mycobacterial ATP/ADP levels with spatiotemporal resolution. This sensor was utilized for tracking the temporal changes in ATP/ADP upon treatment with the antimycobacterial drugs INH, RIF, and BDQ. The use of this novel tool also revealed that Mtb cells residing in macrophages display lower ATP/ADP levels than do extracellular Mtb cells. Interestingly, Mtb cells residing in activated macrophages possess even lower levels of ATP/ADP. Most importantly, we have established that Mtb cells residing inside phagosomes do not face metabolic stress, while the bacilli residing in autophagosomes and phagolysosomes face metabolic stress. Interestingly, even in activated macrophages, phagosomes may serve as a growth-favoring niche. However, the activation of macrophages promotes the maturation of phagosomes into autophagosomes and phagolysosomes and thus inhibits the growth of intracellular bacilli.

This study describes the development, validation, and utilization of new reporter strains of fast- and slow-growing mycobacteria to monitor ATP/ADP levels. The method described in the present study has several advantages over conventional assays, as it provides details of ATP/ADP levels in a population with resolution to the level of the single cell and possesses the ability to track populations over time. A critical component of this study is the adaptation of the sensor PHR in Mycobacterium. Initial experiments suggested that the 420-nm peak of the PHR sensor is masked by mycobacterial autofluorescence. This led us to fuse PHR with mCherry. The fusion product selectively responds to ATP/ADP and could measure ATP/ADP in fast- and slow-growing species of Mycobacterium. We believe that this new methodology is better than the previous method developed by Maglica and coworkers (26), wherein the ATP sensor ATeam was adapted for measurement of the mycobacterial bioenergetics state (26). However, the usage of ATeam in M. smegmatis requires the deletion of the *fbiC* gene to counter mycobacterial autofluorescence. It is expected that the usage of ATeam in Mtb will also require creating mutant strains with a deletion of the *fbiC* gene, as Mtb also possesses autofluorescence at 420 nm. The *fbiC* gene is involved in the biosynthesis of coenzyme F_420_ (52). Cofactor F_420_ could act as an electron carrier and plays an important role in the bioenergetics of *Archaea*. Cofactor F_420_ is known to protect Mtb cells from ROS (53). Thus, the *fbiC* mutant does not represent an ideal tool for studying mycobacterial physiology. Furthermore, we have used the novel reporter for monitoring the ATP/ADP levels of slow-growing Mtb and fast-growing M. smegmatis. In contrast, the ATeaM1.03^YEMK^ FRET-based sensor was used previously only for measuring ATP/ADP in the fast-growing species M. smegmatis (26). Although in the current study, we have utilized PHR-mCherry for measurement of ATP/ADP, we believe that other next-generation sensors such as QUEEN (22) and iATPSnFRs (23) that respond to ATP alone could also be utilized in the future.

Another interesting finding of this study was that the frontline drug RIF alters Mtb cells’ metabolism *in vitro*. In this study, we have explored the effect of antimycobacterial agents, namely, BDQ, INH, and RIF, on mycobacterial metabolism. These antimycobacterial agents have different killing efficiencies and kill kinetics. INH kills Mtb cells quickly but is primarily effective against replicating mycobacterial cells (54). RIF kills slowly and in a time-dependent manner but kills a larger population of bacilli (54). BDQ kills replicating and nonreplicating cells but is bacteriostatic for 4 days and becomes bactericidal after this time point (42). Importantly, BDQ, being an oxidative phosphorylation inhibitor, works through modulation of metabolism (55, 56). Whether INH and RIF alter the metabolism of Mtb has been largely ignored since these agents are well known to inhibit cell wall synthesis and transcription, respectively. In agreement with these results, we observed that BDQ alters Mtb metabolism by as early as 3 h while RIF alters metabolism after 6 h of treatment. These findings are contrary to the well-described inhibition of transcription by RIF (57). However, another frontline drug, INH, does not interfere with metabolism within 12 h of exposure. It is plausible that the metabolism of Mtb will be altered upon treatment with all antibiotics at later time points. Importantly, we also observed that RIF alters the metabolism of Mtb within 6 h. However, the inhibition of metabolism by RIF early on indicates that the transcription of several housekeeping genes is critical for maintaining an optimal metabolic state. Alternatively, RIF could also perturb mycobacterial metabolism. These observations are in agreement with a previous study suggesting that RIF treatment inhibits respiration (58). However, the molecular details of the mechanism of inhibition of metabolism need to be delineated and will require further studies.

Another interesting observation of this study was heterogeneity in the Mtb cells’ metabolic state *in vitro* and *in vivo*. Such heterogeneity is not captured by conventional approaches, which lack spatial resolution. Utilizing the spatial resolution of the engineered reporter strains, we created an ensemble energetics profile of intracellular Mtb. It is worth pointing out that we observed a “nonuniformity” in the intracellular ATP/ADP levels in several images. In several images, we observed that higher ATP/ADP levels accumulated at one of the poles. The concentration gradient of ATP is very well described in mammalian cells such as liver, kidney, and neuronal cells (59–61). Although spatial ATP heterogeneity within single bacteria has been observed previously (22), it is not well defined. The observation of higher ATP levels at one of the poles seems conceivable in the light of the observation that respiratory complex III and complex IV associate together to form a respirasome-like complex in mycobacterial cells (55, 62, 63). Furthermore, respirasomes are known to physically interact with the oligomeric ATP synthase (64). In the light of these data, such a respiratory complex could localize at a particular subcellular position on the bacterial membrane. But the unequivocal demonstration of such a complex and its subcellular localization in mycobacteria requires more research.

This study’s additional exciting finding was observing reduced ATP/ADP levels in intracellular Mtb compared to extracellular Mtb cells. These observations align with our previous study that suggests a higher level of NADH/NAD^+^ in Mtb residing inside macrophages (31). Notably, the ATP/ADP level further decreases upon the activation of macrophages with IFN-γ. These findings provide an energetics insight into the observation that Mtb cells replicate within naive macrophages, while their growth is retarded inside activated macrophages (65). However, it must be noted that intracellular mycobacteria have much higher bioenergetic heterogeneity than extracellular bacilli. These observations align with the previous view that intracellular Mtb cells exhibit higher metabolic heterogeneity ascertained through NADH/NAD^+^ (31) and intracellular redox heterogeneity as determined by estimating mycothiol redox potential (66).

Another significant outcome of this study is the measurement of ATP/ADP levels of Mtb residing within subcellular compartments, namely, phagosomes, autophagosomes, and phagolysosomes. For the first time, this study reports that Mtb cells residing inside phagosomes maintain relatively high ATP/ADP levels. These observations suggest that phagosomes could represent a replicative niche for intracellular Mtb. On the other hand, Mtb cells living within autophagosomes and phagolysosomes experience a depletion of ATP. Such a depletion of ATP could lead to persister formation in these subvacuolar compartments, suggesting that autophagosomes and phagolysosomes may harbor drug-tolerant bacilli. These findings align with previous observations wherein Mtb residing inside autophagosomes and phagolysosomes faces thiol oxidative stress (66). It is important to note that only a minor fraction of bacterial cells within these compartments display high ATP/ADP levels. It remains unknown how a few bacilli can maintain sufficient ATP/ADP levels in these hostile subcellular compartments. Interestingly, the Mtb cells’ metabolic states within all three compartments remain comparable and are not much affected by the activation of macrophages. However, IFN-γ-mediated macrophage activation effectively reduces bacterial growth by increasing the turnover of phagosome maturation into autophagosomes and phagolysosomes, thereby reducing the overall population with optimal metabolism for replication. These findings point toward a scenario suggesting that intracellular Mtb cells modulate their metabolism to adjust to the particular subcellular niche, with phagosomes allowing sufficient metabolism to maintain high ATP/ADP levels and autophagosomes and phagolysosome representing a niche wherein Mtb metabolism is altered to decrease ATP/ADP levels.

## MATERIALS AND METHODS

### Chemicals and drugs.

All chemicals were purchased from Sigma-Aldrich unless mentioned otherwise. We procured BDQ from MedChem Express. 7H9, 7H10, and 7H11 media were acquired from BD Biosciences.

### Bacterial strains, media, and cell culture conditions.

In this study, we utilized nonpathogenic Msmeg mc^2^155 and pathogenic Mtb H37Rv (ATCC 27294). Both strains were transformed with either pMV762-PHR-mCherry or pMV761-PHR-mCherry using a standard electroporation protocol (67). Transformed bacteria were selected on a 7H10 agar plate containing 10% oleic acid-albumin-dextrose-catalase (O-ADC) and 50 μg/ml of hygromycin B in the case of pMV762-PHR-mCherry and 25 μg/ml kanamycin in the case of pMV761-PHR-mCherry. A single colony of transformed bacteria was inoculated in 7H9 medium supplemented with 10% O-ADC–Tween 80 (0.1%) with an appropriate selection marker. The episomal plasmid (pMV762) and the integrative plasmid (pMV761) utilize the constitutive *hsp60* promoter in mycobacteria.

### Expression and purification of PHR-mCherry.

The PHR-mCherry gene was cloned into the pET-28a (kanamycin resistance) plasmid and then transformed into Rosetta(DE3) cells to express the protein. A single positive colony was selected from plates and incubated overnight at 37°C in 10 ml Luria broth as a primary culture. The log-phase culture at an optical density at 600 nm (OD_600_) of 0.6 was induced with 1 mM isopropyl-β-d-thiogalactopyranoside (IPTG) for 6 h at 24°C. The culture was kept on ice for 30 min and then centrifuged at 6,000 rpm (6,641 relative centrifugal force [rcf]) for 10 min. The pellet was collected and sonicated in protein purification buffer (PPB) containing 50 mM Tris (pH 8.0), 300 mM NaCl, 10% glycerol, and phenylmethylsulfonyl fluoride (PMSF). After sonication, the lysate was centrifuged for 15 min at 13,000 rpm (17,949 rcf) at 4°C. The supernatant was collected, and 10 mM imidazole was added to the supernatant. One milliliter of Ni-nitrilotriacetic acid (NTA) beads was equilibrated with 10 ml of equilibration buffer (PPB and 10 mM imidazole). The lysate was then loaded onto an equilibrated column and incubated at 4°C for 1 h. Beads were then washed to remove unwanted proteins with 20 ml PPB with different imidazole concentrations (10 mM, 20 mM, and 30 mM), and the desired protein was eluted with 250 mM imidazole. Excess imidazole was removed by dialysis.

### Generation of mycobacterial reporter strains.

The ATP/ADP biosensor PHR (25) was modified by adding mCherry at the C terminus of PHR through a linker. The modified sensor of ATP/ADP was named PHR-mCherry. The gene encoding PHR-mCherry was codon optimized for expression in Mycobacterium and synthesized using services from GenScript. Codon-optimized PHR-mCherry was then cloned into the mycobacterial shuttle vectors pMV761 and pMV762 at the NcoI and BstBI restriction sites. The orientation and organization of clones were confirmed by restriction digestion and sequencing.

### Treatments.

ATP/ADP reporter strains of Mycobacterium (Msmeg/Mtb) expressing the PHR-mCherry sensor were cultured in 7H9 medium containing 10% OADC, Tween 80 (0.05 to 0.1%), and 50 μg/ml of hygromycin B in the case of pMV762 or 25 μg/ml kanamycin in the case of pMV761. We treated the log-phase cultures (OD_600_ = 0.5 to 0.8) of reporter strains of mycobacteria with different inhibitors/antibiotics. All the stock solutions of inhibitors/antibiotics were prepared in dimethyl sulfoxide (DMSO) unless mentioned otherwise. One milliliter of the log-phase culture was aliquoted into 12-well culture plates. ATP inhibitors/antibiotics were then added to the culture dishes. Fast-growing Msmeg was exposed to inhibitors/antibiotics for 3 h, while slow-growing Mtb was exposed to inhibitors/antibiotics for 3, 6, 12, and 24 h. After incubation, the samples were fixed with 4% paraformaldehyde (PFA) for 15 min and then washed three times with 1× phosphate-buffered saline (PBS). The samples were mounted on glass slides after mixing with Slow Fade. Slides were subjected to confocal microscopy. The following antibiotics/inhibitors were used: isoniazid (0.06 μg/ml), RIF (0.03 μg/ml), BDQ (0.35 μM), and DCCD (100 μM). The MICs of these antibiotics were 0.06 μg/ml for isoniazid, 0.03 μg/ml for RIF, and 0.7 μM for BDQ. Mtb cells in the exponential phase (OD of 0.6) were also exposed to 5 mM arsenate (catalogue no. RM2438-250G; Hi-Media) (CAS no. 10048-95-0) for 3 h.

### Fluorimetric assay.

Two-hundred-microliter aliquots of mid-log-phase (OD_600_ = 0.5 to 0.8) cultures of the reporter strain were added to the wells of 96-well flat-bottom plates. These cultures were then exposed to the above-specified concentrations of inhibitors, and the fluorescence spectra were recorded using a BioTek hybrid fluorimeter. The excitation wavelengths were 500 nm and 587 nm. The emission was recorded at 525 nm and 610 nm. The ratio of the fluorescence at an excitation wavelength 500 nm to that at 587 nm (500/587 ratio) was used to compute the ATP/ADP ratio.

### Estimation of ATP of the bacterial population by a luciferase assay.

Mtb cells (OD_600_ = 1.0) were incubated with BDQ (0.35 μM) and DCCD (100 μM) along with the control (DMSO) for 3 h, and equal volumes of cells from all samples in triplicates were then taken and centrifuged at 6,000 rpm (6,641 rcf) for 5 min, followed by washing of cells with Tris-EDTA (TE) buffer. Five hundred microliters of TE buffer was added to the bacterial pellets. Cells were then lysed with a bead beater (8 cycles, with each consisting of a 30-s pulse at a speed of 6.5 m/s^2^ followed by a 5-min incubation at ice). The cell lysate was centrifuged at 10,000 rpm (9,384 rcf) for 10 min. ATP was estimated using an ATP bioluminescence assay kit (catalogue no. 11699695001; CLS II) according to the manufacturer’s instructions. Briefly, 50 μl of the supernatant was mixed with reaction buffer containing luciferase in 96-well plates, and bioluminescence was measured. Data were plotted using GraphPad Prism software.

### CLSM imaging.

Log-phase (OD_600_ = 0.5 to 0.8) cultures of ATP/ADP reporter strains of mycobacteria were treated with different inhibitors or antibiotics for the specified time intervals. Aliquots were drawn from the cultures and fixed with 4% PFA for 15 min, washed three times with PBS, and resuspended in PBS. Fixed cells were mounted on coverslips. Images were then acquired using the Nikon A1R confocal laser scanning microscope. Images were captured at a resolution of either 512 by 512 or 1,024 by 1,024 pixels with a pinhole of 1.0 by exciting the fluorophore with a 488-nm laser and collecting the emission using a 525/50-nm filter. Also, images were captured by exciting the fluorophore with a 561-nm laser and collecting the emission using a 625/50-nm filter. We utilized sequential scanning with different lasers to prevent bleed-through between fluorescence channels. Both lasers (488 and 561 nm) were used in a sequential manner (sequential line scanning) to reduce cross talk and florescence bleed among different channels. A 60× oil or 100× oil lens objective was utilized for scanning images using the following settings: laser selection channel 1 (Ch1) (488-nm laser), HV-135, offset of 0, and laser power of 8, and Ch2 (561-nm laser), HV-120, offset of 0, and laser power of 4. For immunostaining, the third channel, Ch3 (647-nm laser), was used with the following settings: HV-120, offset of 0, and laser power of 6. These parameters were kept largely constant during all the experiments. The scan size was 512, while the scan speed was 1/2, and the average scan count was 4. This helps in reducing noise, and a digital zoom of 2.5 was used. Before capturing the images, pixel saturation indicators were used to adjust the laser’s sensitivity. Parallel to these, ratiometric images (488/561 nm) were captured, and the ratio scale was generally kept fixed.

### Ratiometric analysis.

After acquiring the original images, ratiometric images were created by dividing the image captured at 488 nm with the images captured at a 561-nm excitation wavelength (green/red). NIS-elements software was used for the analysis of the ATP/ADP ratio of the bacterial population. For this, the “ratio properties” option of NIS-elements was used. We selected the 488-nm channel (for PHR) as the numerator and the 561-nm channel (mCherry) as the denominator. We adjusted the ratio range as a minimum (min) of 0 to maxima (max) of 1, 1.5, 2, 2.5, and 3 depending upon the color of the ratiometric bacterial population images. Next, under “analysis control,” we selected “automated measurement” and “automated measurement results.” In “automated measurement,” we chose the “auto detect” function of the software. After that, bacteria were selected one by one. Next, we choose “keep update measurement” in “automated measurement results.” The data were then exported to an Excel file with all information such as the fluorescence intensity of PHR and mCherry and the mean ratio (PHR/mCherry) of a single bacterium in a bacterial population. The mean ratio for 100 bacteria from each sample was plotted as a scatterplot in GraphPad Prism software. Bacteria showing unusually high mean ratios (>4.0) were excluded. The background of images was not subtracted during analysis and also in representation images. These ratiometric images depict the ATP/ADP ratio inside the bacterial cells. For each experiment, ∼100 bacteria were randomly selected from each sample. The bacterial cell population was binned into populations with low, moderate, and high green/red ratios, depicting bacterial populations with low, moderate, and high ATP/ADP levels. ATP/ADP levels for the control and treatments were compared by a color scale (with violet representing less ATP/ADP and red representing high ATP/ADP levels). The ratiometric intensity was set using the control. This also helped to fix the cutoff for binning bacterial cells into populations with low, moderate, and high ATP/ADP levels. The same settings were used to analyze the bacterial population in treated samples (test samples). Specific antibodies for EEA1, LC3, and LAMP1 were used for detecting bacilli located in early phagosomes, autophagosomes, and phagolysosomes, respectively.

### Infection and immunostaining.

A total of 0.25 million macrophages were seeded on coverslips in 12-well plates. Macrophages were activated with IFN-γ (400 U/ml) or LPS (50 ng/ml) (L2630-10MG from Sigma) for 3 h. After activation, macrophages were infected with the ATP/ADP reporter strain of Mtb H37Rv at a multiplicity of infection (MOI) of 1:10 for 3 h. Next, cells were washed three times with warm PBS to remove the extracellular H37Rv. At 3 h postinfection (p.i.), cells were fixed with 4% PFA for 15 min, followed by three washes with PBS. These cells were then analyzed by confocal microscopy directly or processed for immunostaining. For immunostaining, cells were permeabilized with digitonin (50 μg/ml) for 5 min, followed by three washes with PBS. The cells were incubated in 3% bovine serum albumin (BSA) (blocking buffer) for 1 h and then incubated with primary antibody for 2 h at room temperature. The anti-EEA1 antibody was used for labeling early endosomes/phagosomes (referred here as phagosomes). Anti-LAMP1 and anti-LC3 antibodies were used for labeling the phagolysosomes and autophagosomes, respectively. After 2 h of incubation with primary antibodies, cells were washed three times with PBS. Cells were then incubated with the secondary antibody (dissolved in 3% BSA) for 1 h. Cells were then washed three times with PBS to remove residual antibodies. The coverslips were mounted onto a slide using a mounting reagent (Slow Fade) and then sealed with nail polish, and the ATP/ADP levels were analyzed by using a confocal microscope.

### Antibiotic treatments of intracellular bacteria.

RAW 264.7 macrophages were seeded on coverslips in 12-well plates in triplicates for each drug. Macrophages were infected with the ATP/ADP reporter strain at an MOI of 1:10 for 3 h. Cells were washed three times with warm PBS to remove extracellular bacteria. One milliliter of antibiotic-free DMEM supplemented with 10% fetal bovine serum (FBS) was added, and the cells were then treated with different antibiotics at 5× MIC for 6 h. The samples were fixed with 4% PFA at room temperature for 15 min and then washed three times with PBS. The coverslips were mounted onto the slide using a mounting reagent (Slow Fade), followed by confocal microscopy and analysis.

### Statistical analysis.

The data shown are representative of results from at least three independent experiments performed with technical triplicates. Data are presented as means ± standard errors of the means (SEM), and the significance was calculated using a two-tailed or one-tailed unpaired *t* test.

## References

[1] Hardie DG, Ross FA, Hawley SA. 2012. AMPK: a nutrient and energy sensor that maintains energy homeostasis. Nat Rev Mol Cell Biol 13:251–262. doi:10.1038/nrm3311. 22436748 PMC5726489

[2] Carling D, Mayer FV, Sanders MJ, Gamblin SJ. 2011. AMP-activated protein kinase: nature’s energy sensor. Nat Chem Biol 7:512–518. doi:10.1038/nchembio.610. 21769098

[3] Koebmann BJ, Westerhoff HV, Snoep JL, Nilsson D, Jensen PR. 2002. The glycolytic flux in Escherichia coli is controlled by the demand for ATP. J Bacteriol 184:3909–3916. doi:10.1128/jb.184.14.3909-3916.2002. 12081962 PMC135175

[4] Jensen PR, Michelsen O. 1992. Carbon and energy metabolism of atp mutants of Escherichia coli. J Bacteriol 174:7635–7641. doi:10.1128/jb.174.23.7635-7641.1992. 1447134 PMC207475

[5] Gabriel JL, Zervos PR, Plaut GW. 1986. Activity of purified NAD-specific isocitrate dehydrogenase at modulator and substrate concentrations approximating conditions in mitochondria. Metabolism 35:661–667. doi:10.1016/0026-0495(86)90175-7. 3724458

[6] Metallo CM, Vander Heiden MG. 2013. Understanding metabolic regulation and its influence on cell physiology. Mol Cell 49:388–398. doi:10.1016/j.molcel.2013.01.018. 23395269 PMC3569837

[7] Boshoff HI, Barry CE, III. 2005. Tuberculosis—metabolism and respiration in the absence of growth. Nat Rev Microbiol 3:70–80. doi:10.1038/nrmicro1065. 15608701

[8] Kumar A, Toledo JC, Patel RP, Lancaster JR, Jr, Steyn AJ. 2007. Mycobacterium tuberculosis DosS is a redox sensor and DosT is a hypoxia sensor. Proc Natl Acad Sci U S A 104:11568–11573. doi:10.1073/pnas.0705054104. 17609369 PMC1906723

[9] Ohno H, Zhu G, Mohan VP, Chu D, Kohno S, Jacobs WR, Jr, Chan J. 2003. The effects of reactive nitrogen intermediates on gene expression in Mycobacterium tuberculosis. Cell Microbiol 5:637–648. doi:10.1046/j.1462-5822.2003.00307.x. 12925133

[10] Betts JC, Lukey PT, Robb LC, McAdam RA, Duncan K. 2002. Evaluation of a nutrient starvation model of Mycobacterium tuberculosis persistence by gene and protein expression profiling. Mol Microbiol 43:717–731. doi:10.1046/j.1365-2958.2002.02779.x. 11929527

[11] Sherman DR, Voskuil M, Schnappinger D, Liao R, Harrell MI, Schoolnik GK. 2001. Regulation of the Mycobacterium tuberculosis hypoxic response gene encoding alpha-crystallin. Proc Natl Acad Sci U S A 98:7534–7539. doi:10.1073/pnas.121172498. 11416222 PMC34703

[12] Andries K, Verhasselt P, Guillemont J, Gohlmann HW, Neefs JM, Winkler H, Van Gestel J, Timmerman P, Zhu M, Lee E, Williams P, de Chaffoy D, Huitric E, Hoffner S, Cambau E, Truffot-Pernot C, Lounis N, Jarlier V. 2005. A diarylquinoline drug active on the ATP synthase of Mycobacterium tuberculosis. Science 307:223–227. doi:10.1126/science.1106753. 15591164

[13] Rao SP, Alonso S, Rand L, Dick T, Pethe K. 2008. The protonmotive force is required for maintaining ATP homeostasis and viability of hypoxic, nonreplicating Mycobacterium tuberculosis. Proc Natl Acad Sci U S A 105:11945–11950. doi:10.1073/pnas.0711697105. 18697942 PMC2575262

[14] Rajendran M, Dane E, Conley J, Tantama M. 2016. Imaging adenosine triphosphate (ATP). Biol Bull 231:73–84. doi:10.1086/689592. 27638696 PMC5063237

[15] Conlon BP, Rowe SE, Brown Gandt A, Nuxoll AS, Donegan NP, Zalis EA, Clair G, Adkins JN, Cheung AL, Lewis K. 2016. Persister formation in Staphylococcus aureus is associated with ATP depletion. Nat Microbiol 1:16051. doi:10.1038/nmicrobiol.2016.51. PMC4932909 27572649

[16] Shan Y, Brown Gandt A, Rowe SE, Deisinger JP, Conlon BP, Lewis K. 2017. ATP-dependent persister formation in Escherichia coli. mBio 8:e02267-16. doi:10.1128/mBio.02267-16. 28174313 PMC5296605

[17] Kennedy HJ, Pouli AE, Ainscow EK, Jouaville LS, Rizzuto R, Rutter GA. 1999. Glucose generates sub-plasma membrane ATP microdomains in single islet beta-cells. Potential role for strategically located mitochondria. J Biol Chem 274:13281–13291. doi:10.1074/jbc.274.19.13281. 10224088

[18] Bell CJ, Manfredi G, Griffiths EJ, Rutter GA. 2007. Luciferase expression for ATP imaging: application to cardiac myocytes. Methods Cell Biol 80:341–352. doi:10.1016/S0091-679X(06)80017-8. 17445703

[19] Ideguchi Y, Oshikoshi Y, Ryo M, Motoki S, Kuwano T, Tezuka T, Aoki S. 2016. Posttranslationally caused bioluminescence burst of the Escherichia coli luciferase reporter strain. Arch Microbiol 198:35–41. doi:10.1007/s00203-015-1165-5. 26506945

[20] Maghami P, Ranjbar B, Hosseinkhani S, Ghasemi A, Moradi A, Gill P. 2010. Relationship between stability and bioluminescence color of firefly luciferase. Photochem Photobiol Sci 9:376–383. doi:10.1039/b9pp00161a. 20221465

[21] Imamura H, Nhat KP, Togawa H, Saito K, Iino R, Kato-Yamada Y, Nagai T, Noji H. 2009. Visualization of ATP levels inside single living cells with fluorescence resonance energy transfer-based genetically encoded indicators. Proc Natl Acad Sci U S A 106:15651–15656. doi:10.1073/pnas.0904764106. 19720993 PMC2735558

[22] Yaginuma H, Kawai S, Tabata KV, Tomiyama K, Kakizuka A, Komatsuzaki T, Noji H, Imamura H. 2014. Diversity in ATP concentrations in a single bacterial cell population revealed by quantitative single-cell imaging. Sci Rep 4:6522. doi:10.1038/srep06522. 25283467 PMC4185378

[23] Lobas MA, Tao R, Nagai J, Kronschlager MT, Borden PM, Marvin JS, Looger LL, Khakh BS. 2019. A genetically encoded single-wavelength sensor for imaging cytosolic and cell surface ATP. Nat Commun 10:711. doi:10.1038/s41467-019-08441-5. 30755613 PMC6372613

[24] Berg J, Hung YP, Yellen G. 2009. A genetically encoded fluorescent reporter of ATP:ADP ratio. Nat Methods 6:161–166. doi:10.1038/nmeth.1288. 19122669 PMC2633436

[25] Tantama M, Martinez-Francois JR, Mongeon R, Yellen G. 2013. Imaging energy status in live cells with a fluorescent biosensor of the intracellular ATP-to-ADP ratio. Nat Commun 4:2550. doi:10.1038/ncomms3550. 24096541 PMC3852917

[26] Maglica Z, Ozdemir E, McKinney JD. 2015. Single-cell tracking reveals antibiotic-induced changes in mycobacterial energy metabolism. mBio 6:e02236-14. doi:10.1128/mBio.02236-14. 25691591 PMC4338811

[27] Patino S, Alamo L, Cimino M, Casart Y, Bartoli F, Garcia MJ, Salazar L. 2008. Autofluorescence of mycobacteria as a tool for detection of Mycobacterium tuberculosis. J Clin Microbiol 46:3296–3302. doi:10.1128/JCM.02183-07. 18836064 PMC2566091

[28] Pieters J. 2008. Mycobacterium tuberculosis and the macrophage: maintaining a balance. Cell Host Microbe 3:399–407. doi:10.1016/j.chom.2008.05.006. 18541216

[29] Hung YP, Albeck JG, Tantama M, Yellen G. 2011. Imaging cytosolic NADH-NAD(+) redox state with a genetically encoded fluorescent biosensor. Cell Metab 14:545–554. doi:10.1016/j.cmet.2011.08.012. 21982714 PMC3190165

[30] Bhat SA, Iqbal IK, Kumar A. 2018. Quantification of the metabolic heterogeneity in mycobacterial cells through the measurement of the NADH/NAD+ ratio using a genetically encoded sensor. Methods Mol Biol 1745:261–275. doi:10.1007/978-1-4939-7680-5_14. 29476473

[31] Bhat SA, Iqbal IK, Kumar A. 2016. Imaging the NADH:NAD(+) homeostasis for understanding the metabolic response of Mycobacterium to physiologically relevant stresses. Front Cell Infect Microbiol 6:145. doi:10.3389/fcimb.2016.00145. 27878107 PMC5099167

[32] Meier T, Matthey U, von Ballmoos C, Vonck J, Krug von Nidda T, Kuhlbrandt W, Dimroth P. 2003. Evidence for structural integrity in the undecameric c-rings isolated from sodium ATP synthases. J Mol Biol 325:389–397. doi:10.1016/s0022-2836(02)01204-4. 12488103

[33] Steyn AJ, Joseph J, Bloom BR. 2003. Interaction of the sensor module of Mycobacterium tuberculosis H37Rv KdpD with members of the Lpr family. Mol Microbiol 47:1075–1089. doi:10.1046/j.1365-2958.2003.03356.x. 12581360

[34] Lee MH, Pascopella L, Jacobs WR, Jr, Hatfull GF. 1991. Site-specific integration of mycobacteriophage L5: integration-proficient vectors for Mycobacterium smegmatis, Mycobacterium tuberculosis, and bacille Calmette-Guerin. Proc Natl Acad Sci U S A 88:3111–3115. doi:10.1073/pnas.88.8.3111. 1901654 PMC51395

[35] Moore SA, Moennich DM, Gresser MJ. 1983. Synthesis and hydrolysis of ADP-arsenate by beef heart submitochondrial particles. J Biol Chem 258:6266–6271. doi:10.1016/S0021-9258(18)32402-5. 6853484

[36] Gresser MJ. 1981. ADP-arsenate. Formation by submitochondrial particles under phosphorylating conditions. J Biol Chem 256:5981–5983. doi:10.1016/S0021-9258(19)69115-5. 7240187

[37] Bald D, Villellas C, Lu P, Koul A. 2017. Targeting energy metabolism in Mycobacterium tuberculosis, a new paradigm in antimycobacterial drug discovery. mBio 8:e00272-17. doi:10.1128/mBio.00272-17. 28400527 PMC5388804

[38] Black PA, Warren RM, Louw GE, van Helden PD, Victor TC, Kana BD. 2014. Energy metabolism and drug efflux in Mycobacterium tuberculosis. Antimicrob Agents Chemother 58:2491–2503. doi:10.1128/AAC.02293-13. 24614376 PMC3993223

[39] Cook GM, Hards K, Vilcheze C, Hartman T, Berney M. 2014. Energetics of respiration and oxidative phosphorylation in mycobacteria. Microbiol Spectr 2:MGM2-0015-2013. doi:10.1128/microbiolspec.MGM2-0015-2013. PMC4205543 25346874

[40] Montezano D, Meek L, Gupta R, Bermudez LE, Bermudez JC. 2015. Flux balance analysis with objective function defined by proteomics data—metabolism of Mycobacterium tuberculosis exposed to mefloquine. PLoS One 10:e0134014. doi:10.1371/journal.pone.0134014. 26218987 PMC4517854

[41] Schubert OT, Ludwig C, Kogadeeva M, Zimmermann M, Rosenberger G, Gengenbacher M, Gillet LC, Collins BC, Rost HL, Kaufmann SH, Sauer U, Aebersold R. 2015. Absolute proteome composition and dynamics during dormancy and resuscitation of Mycobacterium tuberculosis. Cell Host Microbe 18:96–108. doi:10.1016/j.chom.2015.06.001. 26094805

[42] Koul A, Vranckx L, Dhar N, Gohlmann HW, Ozdemir E, Neefs JM, Schulz M, Lu P, Mortz E, McKinney JD, Andries K, Bald D. 2014. Delayed bactericidal response of Mycobacterium tuberculosis to bedaquiline involves remodelling of bacterial metabolism. Nat Commun 5:3369. doi:10.1038/ncomms4369. 24569628 PMC3948051

[43] Boshoff HI, Myers TG, Copp BR, McNeil MR, Wilson MA, Barry CE, III. 2004. The transcriptional responses of Mycobacterium tuberculosis to inhibitors of metabolism: novel insights into drug mechanisms of action. J Biol Chem 279:40174–40184. doi:10.1074/jbc.M406796200. 15247240

[44] Salim T, Sershen CL, May EE. 2016. Investigating the role of TNF-alpha and IFN-gamma activation on the dynamics of iNOS gene expression in LPS stimulated macrophages. PLoS One 11:e0153289. doi:10.1371/journal.pone.0153289. 27276061 PMC4898755

[45] Nathan CF, Prendergast TJ, Wiebe ME, Stanley ER, Platzer E, Remold HG, Welte K, Rubin BY, Murray HW. 1984. Activation of human macrophages. Comparison of other cytokines with interferon-gamma. J Exp Med 160:600–605. doi:10.1084/jem.160.2.600. 6206183 PMC2187443

[46] Schaible UE, Sturgill-Koszycki S, Schlesinger PH, Russell DG. 1998. Cytokine activation leads to acidification and increases maturation of Mycobacterium avium-containing phagosomes in murine macrophages. J Immunol 160:1290–1296. 9570546

[47] Jensen K, Gallagher IJ, Johnston N, Welsh M, Skuce R, Williams JL, Glass EJ. 2018. Variation in the early host-pathogen interaction of bovine macrophages with divergent Mycobacterium bovis strains in the United Kingdom. Infect Immun 86:e00385-17. doi:10.1128/IAI.00385-17. 29263113 PMC5820943

[48] Woo M, Wood C, Kwon D, Park KP, Fejer G, Delorme V. 2018. Mycobacterium tuberculosis infection and innate responses in a new model of lung alveolar macrophages. Front Immunol 9:438. doi:10.3389/fimmu.2018.00438. 29593716 PMC5858468

[49] Gorvel JP, Chavrier P, Zerial M, Gruenberg J. 1991. rab5 controls early endosome fusion in vitro. Cell 64:915–925. doi:10.1016/0092-8674(91)90316-q. 1900457

[50] Chen JW, Murphy TL, Willingham MC, Pastan I, August JT. 1985. Identification of two lysosomal membrane glycoproteins. J Cell Biol 101:85–95. doi:10.1083/jcb.101.1.85. 2409098 PMC2113627

[51] Kabeya Y, Mizushima N, Yamamoto A, Oshitani-Okamoto S, Ohsumi Y, Yoshimori T. 2004. LC3, GABARAP and GATE16 localize to autophagosomal membrane depending on form-II formation. J Cell Sci 117:2805–2812. doi:10.1242/jcs.01131. 15169837

[52] Choi KP, Kendrick N, Daniels L. 2002. Demonstration that fbiC is required by Mycobacterium bovis BCG for coenzyme F(420) and FO biosynthesis. J Bacteriol 184:2420–2428. doi:10.1128/jb.184.9.2420-2428.2002. 11948155 PMC134996

[53] Gurumurthy M, Rao M, Mukherjee T, Rao SP, Boshoff HI, Dick T, Barry CE, III, Manjunatha UH. 2013. A novel F(420)-dependent anti-oxidant mechanism protects Mycobacterium tuberculosis against oxidative stress and bactericidal agents. Mol Microbiol 87:744–755. doi:10.1111/mmi.12127. 23240649 PMC3567243

[54] de Steenwinkel JE, de Knegt GJ, ten Kate MT, van Belkum A, Verbrugh HA, Kremer K, van Soolingen D, Bakker-Woudenberg IA. 2010. Time-kill kinetics of anti-tuberculosis drugs, and emergence of resistance, in relation to metabolic activity of Mycobacterium tuberculosis. J Antimicrob Chemother 65:2582–2589. doi:10.1093/jac/dkq374. 20947621

[55] Bajeli S, Baid N, Kaur M, Pawar GP, Chaudhari VD, Kumar A. 2020. Terminal respiratory oxidases: a targetables vulnerability of mycobacterial bioenergetics? Front Cell Infect Microbiol 10:589318. doi:10.3389/fcimb.2020.589318. 33330134 PMC7719681

[56] Iqbal IK, Bajeli S, Akela AK, Kumar A. 2018. Bioenergetics of Mycobacterium: an emerging landscape for drug discovery. Pathogens 7:24. doi:10.3390/pathogens7010024. PMC5874750 29473841

[57] Sippel A, Hartmann G. 1968. Mode of action of rafamycin on the RNA polymerase reaction. Biochim Biophys Acta 157:218–219. doi:10.1016/0005-2787(68)90286-4. 4868249

[58] Lobritz MA, Belenky P, Porter CB, Gutierrez A, Yang JH, Schwarz EG, Dwyer DJ, Khalil AS, Collins JJ. 2015. Antibiotic efficacy is linked to bacterial cellular respiration. Proc Natl Acad Sci U S A 112:8173–8180. doi:10.1073/pnas.1509743112. 26100898 PMC4500273

[59] Jones DP. 1986. Intracellular diffusion gradients of O2 and ATP. Am J Physiol 250:C663–C675. doi:10.1152/ajpcell.1986.250.5.C663. 3010727

[60] Aw TY, Jones DP. 1985. ATP concentration gradients in cytosol of liver cells during hypoxia. Am J Physiol 249:C385–C392. doi:10.1152/ajpcell.1985.249.5.C385. 2998197

[61] Jones DP, Mason HS. 1978. Gradients of O2 concentration in hepatocytes. J Biol Chem 253:4874–4880. doi:10.1016/S0021-9258(17)34627-6. 209020

[62] Wiseman B, Nitharwal RG, Fedotovskaya O, Schafer J, Guo H, Kuang Q, Benlekbir S, Sjostrand D, Adelroth P, Rubinstein JL, Brzezinski P, Hogbom M. 2018. Structure of a functional obligate complex III2IV2 respiratory supercomplex from Mycobacterium smegmatis. Nat Struct Mol Biol 25:1128–1136. doi:10.1038/s41594-018-0160-3. 30518849

[63] Gong H, Li J, Xu A, Tang Y, Ji W, Gao R, Wang S, Yu L, Tian C, Li J, Yen H-Y, Man Lam S, Shui G, Yang X, Sun Y, Li X, Jia M, Yang C, Jiang B, Lou Z, Robinson CV, Wong L-L, Guddat LW, Sun F, Wang Q, Rao Z. 2018. An electron transfer path connects subunits of a mycobacterial respiratory supercomplex. Science 362:eaat8923. doi:10.1126/science.aat8923. 30361386

[64] Wittig I, Schagger H. 2009. Supramolecular organization of ATP synthase and respiratory chain in mitochondrial membranes. Biochim Biophys Acta 1787:672–680. doi:10.1016/j.bbabio.2008.12.016. 19168025

[65] Rook GA, Steele J, Ainsworth M, Champion BR. 1986. Activation of macrophages to inhibit proliferation of Mycobacterium tuberculosis: comparison of the effects of recombinant gamma-interferon on human monocytes and murine peritoneal macrophages. Immunology 59:333–338. 3098676 PMC1453207

[66] Bhaskar A, Chawla M, Mehta M, Parikh P, Chandra P, Bhave D, Kumar D, Carroll KS, Singh A. 2014. Reengineering redox sensitive GFP to measure mycothiol redox potential of Mycobacterium tuberculosis during infection. PLoS Pathog 10:e1003902. doi:10.1371/journal.ppat.1003902. 24497832 PMC3907381

[67] Goude R, Roberts DM, Parish T. 2015. Electroporation of mycobacteria. Methods Mol Biol 1285:117–130. doi:10.1007/978-1-4939-2450-9_7. 25779313

